# The efficacy and safety of PD-1/PD-L1 inhibitors in combination with chemotherapy as a first-line treatment for unresectable, locally advanced, HER2-negative gastric or gastroesophageal junction cancer: a meta-analysis of randomized controlled trials

**DOI:** 10.3389/fimmu.2025.1566939

**Published:** 2025-03-26

**Authors:** Wenji Pu, Shasha Li, Jinliang Zhang, Jijie Huang, Jishi Li, Yong Jiang, Zhiyuan Xu, Fan Yi, Yuling Lan, Qin Xiao, Wenqi Chen, Jing Jin

**Affiliations:** ^1^ Department of Clinical Oncology, The University of Hong Kong-Shenzhen Hospital, Shenzhen, China; ^2^ National Cancer Center/National Clinical Research Center for Cancer/Cancer Hospital & Shenzhen Hospital, Chinese Academy of Medical Sciences and Peking Union Medical College, Shenzhen, China; ^3^ Medical Department of Shenzhen University/General Hospital of Shenzhen University/Academy of Clinical Medicine of Shenzhen University, Shenzhen, China; ^4^ State Key Laboratory of Molecular Oncology and Department of Radiation Oncology, National Cancer Center/National Clinical Research Center for Cancer/Cancer Hospital, Chinese Academy of Medical Sciences and Peking Union Medical College, Beijing, China

**Keywords:** advanced gastroesophageal cancer, immune check points, PD-1/PD-L1 inhibitors, chemotherapy, gastroesophageal adenocarcinoma, gastric adenocarcinoma, overall survival, meta-analysis

## Abstract

**Background:**

Immune checkpoint inhibitors (ICIs) plus fluorouracil-based chemotherapy (Chemo) have been approved as an initial treatment strategy for metastatic or recurrent human epidermal growth factor receptor 2 (HER2)-negative gastric cancer (GC) or gastroesophageal junction cancer (GEJC). However, since programmed cell death protein-1 (PD-1) or its ligand 1 (PD-L1) inhibitors have just recently been investigated for the treatment of unresectable GC/GEJC, there is ongoing debate regarding their safety and effectiveness for prespecified subgroups. The purpose of this research is to establish a foundation toward stratified decision-making by methodically assessing the merits and drawbacks of PD-1/PD-L1 inhibitors combined with chemo in the clinical utilization of advanced HER2-negative GC/GEJC according to certain prominent large-scale randomized controlled trials (RCTs). In addition, we limitedly explored the favorable short-term efficacy of PD-1/CTLA-4 bispecific antibodies for the above-mentioned tumors.

**Methods:**

The researchers retrieved several databases, including PubMed, Embase, Web of Science, ClinicalTrials.gov, and the Cochrane Library, to collect all the relevant literature published since the establishment of the databases until October 30, 2024, and then screened to determine the qualified literature and extracted the relevant information. We only included RCTs for PD-1/PD-L1 inhibitors with or without chemo in advanced GC or GEJC. The primary endpoints were overall survival (OS), progression-free survival (PFS), and objective response rate (ORR). A subgroup analysis for the median overall survival (mOS) was conducted for the following variables: microsatellite instability (MSI) status, PD-L1 expression, combined positive scores (CPS), metastasis status, and primary tumor location. When moderate heterogeneity was found, a random-effect model was applied. The outcome indicators were then statistically analyzed, taking advantage of Review Manager 5.4. Hazard ratio (HR) and risk ratio (RR) were selected as the effect values for statistical analysis.

**Results:**

A total of 7 eligible RCTs and 6537 participants were included in this meta-analysis. Combining PD-1/PD-L1 inhibitors with chemo significantly improved patients’ OS compared with chemo alone, especially in the tumor cell PD-L1 expression ≥ 1% [HR = 0.62, 95% CI (0.48, 0.81); a p-value = 0.0004], PD-L1 CPS ≥ 10 [HR = 0.66, 95% CI (0.57, 0.77); a p-value < 0.00001], and MSI-H subgroups [HR = 0.40, 95% CI (0.28, 0.59); a p-value < 0.00001]. Moreover, distinct primary tumor location (GC or GEJC) and the presence of liver metastases could also benefit from the additive or sustained effect of anti-cancer chemo-immunotherapy.

**Conclusion:**

For patients with advanced HER2-negative GC/GEJC, PD-1/PD-L1 inhibitors in combination with chemo have almost demonstrated consistent synergistic anti-tumor benefits to survival outcomes when compared to chemo alone. However, the subgroup analysis in this meta-study revealed that neither PD-L1 expression level nor MSI status could fully predict the efficacy of the dual treatment model but faced a higher possibility of serious treatment-related adverse events (sTRAEs), particularly in the synchronous therapy arm. Therefore, urging the need for more investigations into the development of collaborative prognostic forecasting models for achieving precise stratification, established harmonized testing standards and methods for PD-L1 expression and positivity, optimal CPS threshold for benefits, as well as alternative molecular biomarkers for the reason that certain indicators alone may not discriminate responders clearly. Lastly, dual anti-therapy might be a useful tactic for the population with low PD-L1 expression in the future.

## Highlights

- For HER2-negative adenocarcinoma, there is still no targeted agent that has been successfully evaluated and proved to prolong survival in addition to the immunization drug. 1L PD-1/PD-L1 inhibitors plus chemo regimens for patients with advanced HER2-negative GC or GEJC are standard of care but limited by study length and inconsistent survival benefits.
- PD-L1 appears to be the best marker of efficacy for GC or GEJC at the present. Neither PD-L1 CPS nor MSI status could fully predict the efficacy of the dual treatment model but was accompanied by higher sTRAEs.
- Harmonization standards of PD-L1 assays and further other associated biomarker studies may be warranted.
- Dual antibody combination chemo regimens from initial findings significantly improve OS benefits in the entire population with advanced HER2-negative sufferers regardless of PD-L1 expression, which needs to be studied further.

## 1 Introduction

Globally, gastric cancer (GC)/gastroesophageal junction cancer (GEJC) ranks fourth in terms of tumor-related mortality and is the fifth most prevalent type of cancer, with these rankings on the upward trend from global cancer statistics in 2022, which is a concerning event (1). More GC/GEJC patients receive their diagnosis with adenocarcinoma at an advanced or metastatic stage due to their neglect of mild symptoms, meaning the prognosis is bleaker, especially in Asia and the Orient than in Europe and North America (2). Furthermore, about half of eligible, partially advanced patients experience localized and regional recurrence, even peritoneal and distant metastasis, after completing radical D2 surgery for GC, unfortunately within 2 years, which offsets the survival gains in the general population to some extent (3–5). Prior to the introduction of immunotherapy, platinum with fluorouracil-based or triplet chemotherapy (chemo) with fluorouracil plus oxaliplatin and docetaxel was the first-line treatment for patients with no human epidermal growth factor receptor 2 (HER2) overexpression or amplification of advanced GC/GEJC. Due to the great spatial and temporal heterogeneity of malignant tumors, patients with advanced GC/GEJC treated with traditional chemo have a median overall survival (mOS) of just 8 months (m), rarely exceeding 1 year (6). The lack of focused selectivity of chemo to tumor cells, chemotherapeutic drug resistance and toxicity, and the challenges of screening the favorable recipients restrict the advantages of traditional regimens (7).

The first successful treatment target for GC/GEJC is the HER2. According to data from earlier large-scale multicenter trials conducted in China, the HER2-overexpressing rate of GC/GEJC patients is between 12% and 13%, but research conducted abroad has found that approximately 20% of patients have HER2 gene amplification or protein overexpression, which may be partly attributed to the variance of the testing specimen handling process and criteria for interpretation (8–10). As for HER2-negative adenocarcinoma, there is still no targeted agent that has been evaluated successfully and proved to prolong survival relative to chemo.

Luckily, immunotherapy has grown quickly in recent years and is now a research hotspot for treating malignancies, offering fresh concepts for clinical interventions in patients with advanced non-HER2-positive GC/GEJC (11–13). In order to boost their own survival rate and evade the immunological response, cancer cells have been shown to increase the expression of proteins involved in the programmed cell death pathway and transmit immunosuppressive signals to the immune system. Conversely, monoclonal antibodies against programmed cell death protein-1 (PD-1) or its ligand 1 (PD-L1) disrupt the binding between them, respectively, which positively regulates T cell activation and promotes tumor cell killing by elicited CD8+ T cells (14). Currently, immune checkpoint inhibitors (ICIs) have progressively advanced from third-line treatment to second-line or even first-line treatment for GC/GEJC. The treatment of advanced GC/GEJC with monoclonal antibodies against PD-1 and PD-L1 through induction of immunogenic cell death has demonstrated remarkable clinical results, obtaining front-line treatment status ultimately and bringing a paradigm shift in the standard of care. So far, several countries, including South Korea and Japan, and the Food and Drug Administration (FDA), have approved the clinical application of anti-PD-1 antibodies, such as nivolumab, pembrolizumab, and sintilimab, plus chemo for this indication, particularly among the PD-L1 combined positive scores (CPS) of ≥5 populations. Additionally, the National Medical Products Administration (NMPA) approved the new drug’s indication for first-line therapy of advanced GC or GEJC on September 30, 2024, given that Cadonilimab (AK104) is an innovation-oriented PD-1/CTLA-4 bispecific antibody that could target both PD-1 and CTLA-4 to greatly improve anti-tumor immune response.

However, the effectiveness and safety of PD-1/PD-L1 inhibitors with chemo in the treatment of advanced GC/GEJC remain debatable owing to their comparatively limited study length and inconsistent survival benefits. The purpose of this investigation is to establish a foundation for clinical decision-making by methodically weighing the pros and cons of ICIs with certain concerning outcome indicators, including overall survival (OS), progression-free survival (PFS), objective response rate (ORR), serious treatment-related adverse events (sTRAEs), and other metrics. Moreover, various indicators for predicting the effectiveness of ICIs therapy in certain gastrointestinal tumors have been discovered to guide clinical practice, such as high microsatellite instability/mismatch repair deficiency (MSI-H/dMMR) or high tumor mutation burden (TMB-H), as well as increased expression level of tumor cell PD-L1. Thus, we also aimed to conduct prespecified subgroup analysis of alternative possible variables potentially related to mOS, aiming to provide a deeper understanding of dual-drug combination regimens and enable stratified precision cancer medicine by summarizing the available evidence.

## 2 Material and methods

### 2.1 Protocols and guidance

We adhered to the Preferred Reporting Items for Systematic Reviews and Meta-Analyses (PRISMA) 2020 statement guidelines, and this study was implemented according to the principles of the PRISMA checklist. And our protocol has been registered with PROSPERO under the following registration ID: CRD42024617955.

### 2.2 Inclusion criteria

According to the Population, Intervention, Comparison, Outcomes, and Study (PICOS) principle, the inclusion criteria were designed as follows: (1) Participants: previously untreated, surgically unresectable HER2-negative locally advanced or metastatic GC/CEJC with definitive pathologic diagnosis and radiological examination; (2) Interventions: the regimen was PD-1/PD-L1 inhibitors plus chemo as a first-line treatment paradigm; (3) Comparisons: the standard chemo regimen (XELOX, FOLFOX, or PF) was adopted; (4) Outcomes: the inclusion of the literature contains at least one of the following indexes: PFS, OS, ORR, sTRAEs ≥3 grade toxicity rate, and characteristics of patients with tumors; (5) Study design: only based on prospective RCTs.

### 2.3 Exclusion criteria

In this analysis, we only focused on full-text published clinical research; conference presentations, abstracts, and other milestone results were not included. We systematically excluded the following publications: (1) studies involving GC/GEJC patients who have developed primary tumors in other sites (including squamous and adenocarcinoma of the esophagus cancer); (2) fuzzy ending indicators; (3) non-prospective RCTs, such as cohort studies, retrospective studies, single-arm clinical trials, and case reports; (4) animal and cellular experiments; (5) unoriginal research, e.g., systematic reviews, study protocols, letters, and expert opinions; (7) repeated publications at different assessment times; (8) trials that lacked a complete set of indicators for extracting the required data.

### 2.4 Outcomes

The primary outcomes were to investigate the effect of immuno-chemotherapy for unresectable or metastatic GC/CEJC on OS, PFS, and ORR. And the mOS was stratified according to the characteristics of the population. Our secondary objective included an analysis of safety indicators, for instance, sTRAEs.

### 2.5 Literature search

We comprehensively searched PubMed, Embase, Web of Science, ClinicalTrials.gov, and the Cochrane Library by using Medical Subject Headings (MeSH) terms of “PD-1 inhibitor,” “PD-L1 inhibitor,” “gastric adenocarcinoma,” “gastroesophageal adenocarcinoma,” and their individual corresponding free terms with combinations of Boolean operators (AND, OR, NOT).

Data search strategies were elaborately designed as follows: (“PD-1 Inhibitor” OR “Programmed Death 1 Inhibitor” OR “Programmed Cell Death Protein 1 Inhibitor” OR “Anti-PD-1” OR “PD-L1 Inhibitor” OR “anti-PD-L1” OR “anti-PD-1/PD-L1” OR “Programmed Death Ligand 1 Inhibitor” OR pembrolizumab OR nivolumab OR avelumab OR sintilimab OR tislelizumab OR camrelizumab OR durvalumab OR atezolizumab OR toripalimab) AND (“gastroesophageal junction” OR “gastro-esophageal junction” OR “gastrooesophageal junction” or “gastro-oesophageal junction” OR esophagogastric OR oesophagogastric OR gastric OR stomach) AND (cancer OR carcinoma OR adenocarcinoma). No limitations were imposed based on country, language, or year of publication. The last search was updated up to October 30, 2024. After screening titles and abstracts, literature selection was conducted by two independent reviewers (Wenji Pu and Shasha Li). Besides, we tracked the reference lists of relevant literature to confirm all eligible studies were included in our meta-analysis. The screening focused on our study endpoints, which mainly encompass OS, as well as the hazard ratio (HR) for the related subgroups.

### 2.6 Assessing the risk of bias of included studies

Applying the Cochrane Collaboration’s risk for bias assessment tool, which addressed the random allocation approach, allocation concealment, blinding, data completeness, selective reporting, and other potential sources of bias (15), we conducted a thorough quality assessment of all included RCTs. Three rating levels—low risk, unclear, and high risk—were assigned to each item. The Review Manager 5.4 software was seized to map the quality evaluation and evaluate the data’s quality. Two researchers (Wenji Pu and Shasha Li) should search the literature and screen it based on inclusion and exclusion criteria. If neither party can agree, they should negotiate a solution; if the disagreement persists, a third reviewer should be consulted to make the final decision.

### 2.7 Data extract

The following information was extracted from each selected publication: first author, country, year of publication, baseline information of patients with neoplasms, interventions and comparisons, survival endings (ORR, OS, and PFS), acute serious toxicity, metastasis status, and PD-L1 expression of tumors. Adverse events and laboratory abnormalities were assessed regularly throughout treatment and up to 30 days after discontinuation (up to 90 days for sTRAEs without anti-cancer therapy) according to the National Cancer Institute Common Terminology Criteria for Adverse Events (CTCAE).

### 2.8 Statistical analysis

If available, RevMan 5.4 software from the Cochrane Collaboration was used for all statistical analysis of the chosen publications. I^2^ values and P values based on χ2 tests were used to evaluate the heterogeneity of the literature data; if P values ≥ 0.1 and I^2^ < 50%, the heterogeneity was deemed minor, and a fixed-effect model was examined; if not, a random-effect model was used. If required, sensitivity or subgroup analysis was used to investigate possible sources of heterogeneity. Hazard ratio (HR) was selected as the effect size for statistical analysis of survival data, and the risk ratio (RR) was selected as the effect value for statistical analysis for bivariate variables. A p-value < 0.05 was regarded as a statistically significant difference.

### 2.9 Subgroup analysis

In the absence of desirable biomarkers, we investigated further with univariate meta-regression in order to differentiate characterized populations that may benefit more from combined therapy and those who may not. The following variables were subjected to subgroup analysis taking advantage of survival data: primary tumor location (GC or GEJC), microsatellite instability status, tumor cell PD-L1 expression, PD-L1 CPS, types of intervention (PD-1/PD-L1 inhibitors with chemo or chemo monotherapy), and the presence of metastatic disease prior to therapy.

### 2.10 Patient and public involvement

Patients were not involved in formulating the study’s design or implementation strategies, nor were they involved in determining the research question or outcome measures. No patients were asked for their opinions on how to draft or interpret the results. Through social media and networks, the findings will be shared with a large audience, including patients, members of the general public, medical professionals, and specialists in this field.

## 3 Results

### 3.1 Literature review

**Figure 1:**
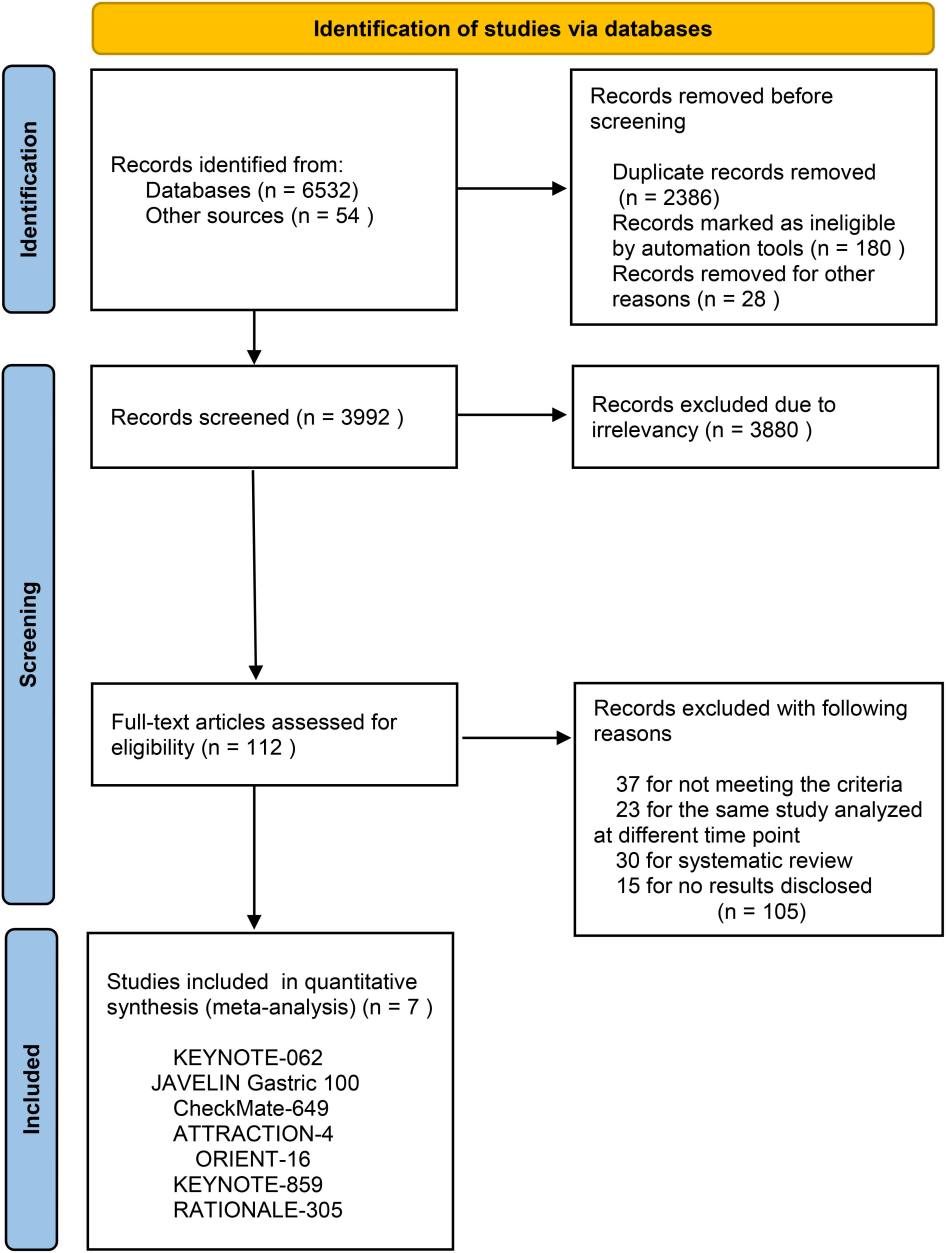
PRISMA 2020 flow diagram for systematic reviews which included searches of databases.

After searching through 6586 pertinent publications, 180 records that were considered ineligible and 2386 duplicates were eliminated. And 3880 publications were deemed irrelevant after all titles and abstracts were examined. 112 possible eligible full-text articles were then evaluated in more detail. 30 publications for a systematic review, 15 for no results provided, 23 for the same study examined at different times, and 37 for not matching the criteria were among the additional 105 records that we eliminated. Lastly, we incorporated 7 RCTs (16–22) into our meta-analysis (**Figure 1** shows the flow diagram). And **Table 1** provided an overview description of the included studies.

**Table 1:** Overview of the included trials.

Trial title	Clinical trial No.	First author	Author country	Study design	Phase	Year of publication	Blindness	Candidates	Region
JAVELIN Gastric 100 (16), Global	NCT02625610	Moehler M	Germany	RCT	Phase III	2021	Open-label	Unresectable, advanced or metastatic adenocarcinoma	North America, Europe, Asia, and the rest of the world (17 countries)
KEYNOTE-062 (17), Global	NCT02494583	Shitara K	Japan	RCT	Phase III	2020	Double-blind	Locally advanced/ unresectable or metastatic GC/GEJC	Europe, North America, Australia, Asia, and the rest of the world (29 countries)
CheckMate 649 (18, 23), Global	NCT02872116	Janjigian Y	USA	RCT	Phase III	2021	Open-label	Unresectable advanced or metastatic adenocarcinoma	Asia, Australia, Europe, North America, and South America (29 countries)
ORIENT-16 (19), China	NCT03745170	Xu J	China	RCT	Phase III	2023	Double-blind	Unresectable locally advanced or metastatic cancer	China (62 hospitals)
ATTRACTION-4 (20), Asia	NCT02746796	Kang Y	Korea	RCT	Phase III	2021	Double-blind	Unresectable advanced or recurrent GC or GEJC	Asia: Japan, South Korea, and Taiwan (146 medical centres)
RATIONALE-305 (21), Global	NCT03777657	Qiu M	China	RCT	Phase III	2024	Double-blind	Locally advanced unresectable or metastatic adenocarcinoma	Asia, Europe, and North America
KEYNOTE-859 (22), Global	NCT03675737	Rha S	Korea	RCT	Phase III	2023	Double-blind	Locally advanced or metastatic adenocarcinoma	Western Europe, Israel, North America, Australia, Asia, and the rest of the world (33 countries)

RCT, Randomized controlled trial; GC, Gastric cancer; GEJC, Gastroesophageal junction cancer.

### 3.2 Characteristics of included study

**Figure 2:**
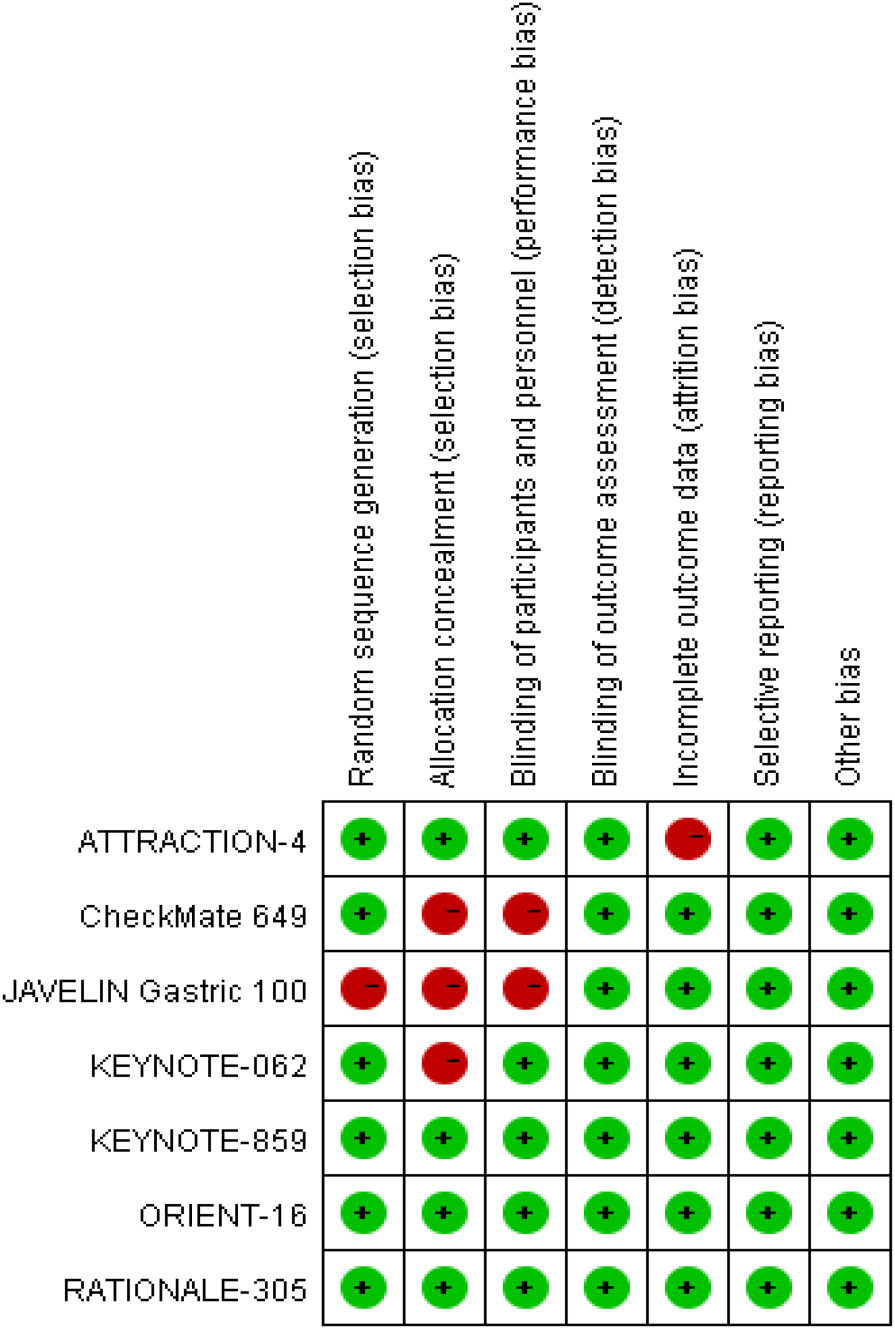
Risk of bias summary according to review authors' judgments about each risk of bias item for the included RCTs.

**Figure 3:**
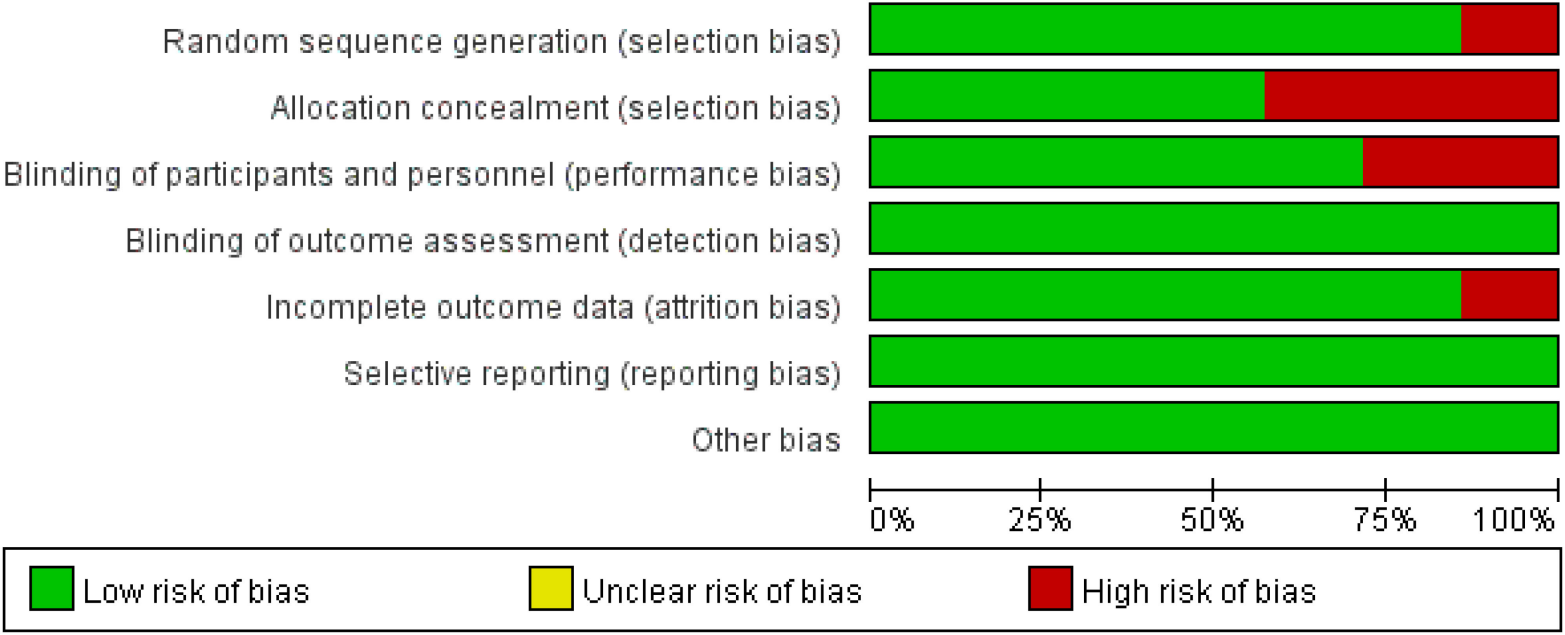
Risk of bias graph.

GC or GEJC patients in the two involved RCTs (16, 18) were informed of the treatment regimen at randomization, thus keeping a secret between the patients and supervising doctors difficult. In addition, the risk of bias assessment and summary is illustrated in **Figures 2**, **3**. A total of 6537 unresectable, advanced, or metastatic tumor patients were randomly assigned to the concurrent/sequential immunochemotherapy group (n = 3275) or the chemo alone group (n = 3262). The neoplasm features and proportions of the included studies were shown in **Table 2**, which were similar between the two arms.

**Table 2:** Summary of the patients' characteristics of the included RCTs.

Trial title	Sample size after randomization (ITT population)	First-line interventions	Patient numbers (Man vs. woman) (%)	Average age (Years)	ECOG status (0 vs. 1) (%)	Tumor location (GC vs. GEJC) (%)	HER2 status	Primary endpoints
JAVELIN Gastric 100 (16), Global	499	Chemo followed by Avelumab (10 mg/kg/2 weeks) maintenance	249 (66% vs. 34%)	62	249 (41% vs. 59%)	249 (70% vs. 30%)	Negative	OS
		Chemo (FOLFOX or CAPEOX)	250 (67% vs. 33%)	61	250 (43% vs. 57%)	250 (72% vs. 28%)		
KEYNOTE-062 (17), Global	507	Pembrolizumab (200 mg/3 weeks) with concurrent chemo	257 (76%/24%)	62	257 (46% vs. 54%)	255 (67% vs. 33%)	Negative	OS and PFS in patients with PD-L1 CPS ≥ 1
		Chemo (Cisplatin plus either fluorouracil or capecitabine)	250 (72%/28%)	63	250 (46% vs. 54%)	248 (73% vs. 27%)		
CheckMate 649 (18, 23), Global	1581	Nivolumab (360 mg/ 3 weeks or 240 mg /2 weeks) plus chemo	789 (68%/32%)	62	788 (41% vs. 59%)	686 (81% vs. 19%)	Negative	OS and PFS in patients with PD-L1 CPS ≥ 5
		CAPEOX or FOLFOX chemo	792 (71%/29%)	61	788 (43% vs. 57%)	684 (81% vs. 19%)		
ORIENT-16 (19), China	650	Sintilimab (3 mg/kg/3 weeks or 200 mg) in combination with XELOX chemo	327 (77%/23%)	62	327 (27% vs. 73%)	326 (82% vs. 18%)	Negative	OS in patients with PD-L1 CPS ≥ 5
		Placebo plus chemo (CAPEOX followed by maintenance dose of capecitabine)	323 (71%/29%)	60	323 (28% vs. 72%)	323 (81% vs. 19%)		
ATTRACTION-4 (20), Asia	724	Nivolumab (360mg/3 weeks) plus SOX or CAPEOX chemo	362 (70%/30%)	64	362 (54% vs. 46%)	266 (89% vs. 11%)	Negative	OS and PFS
		Placebo plus chemo (SOX or CAPEOX)	362 (75%/25%)	65	362 (54% vs. 46%)	271 (88% vs. 12%)		
RATIONALE-305 (21), Global	997	Tislelizumab (200mg/3 weeks) plus CAPEOX or PF chemo	501 (69%/31%)	60	501 (34% vs. 66%)	501 (81% vs. 19%)	Negative	OS and PFS
		Placebo plus chemo (PF or CAPEOX)	496 (70%/30%)	61	496 (31% vs. 69%)	495 (80% vs. 20%)		
KEYNOTE-859 (22), Global	1579	Pembrolizumab (200mg/3 weeks) plus CAPEOX or PF chemo	790 (67%/33%)	61	790 (36% vs. 64%)	789 (81% vs. 19%)	Negative	OS
		Placebo plus chemo (CAPEOX or PF)	789 (69%/31%)	62	789 (38% vs. 62%)	788 (77% vs. 23%)		

RCTs, Randomized controlled trials; ECOG, Eastern Cooperative Oncology Group; ITT, Intention-to-treat; GC, Gastric cancer; GEJC, Gastroesophageal junction cancer; CPS, Combined positive score; OS, Overall survival; PFS, Progression-free survival; Chemo, Chemotherapy; SOX, S-1 plus oxaliplatin; CAPEOX/XELOX, Capecitabine plus oxaliplatin; FOLFOX, leucovorin, fluorouracil, and oxaliplatin; PF, Cisplatin plus fluorouracil.

### 3.3 Quality analysis

Most of the included RCTs (16–22) in our study reported the randomization process and maintained the treatment regimen a secret between the patients and implementers; although 3 RCTs (16–18) did not report the assignment hiding process, the above restriction did not affect the outcome assessment in general.

### 3.4 Primary endpoints: overall survival, progression-free survival, and objective response rate

**Table 3** summarized these findings’ specifics. When available at an intended time point for assessment, efficacy evaluation metrics, including OS, PFS, and ORR, were typically considered crucial references to determine whether or not the main objectives were met within some RCTs.

**Table 3:** Summary of the treatment outcomes and tumor characteristics from the included prospective studies.

Trial title	ICIs drugs	HR for OS (ITT population)	HR for PFS (ITT population)	ORR (%)	sTRAE (%)	MSI-H (%)	GC (%)	Metastasis(%)	Tumor cell PD-L1 expression ≥1%	PD-L1 CPS		
										≥1	≥5	≥10
JAVELIN Gastric 100 (16), global	Avelumab (PD-L1) maintenance	0.91; 95% CI, 0.74-1.11 (mOS: 10.4 m vs. 10.9 m)	1.04; 95% CI, 0.85-1.28 (mPFS: 3.2 m vs. 4.4 m)	13% (13/249)	8% (19/243)	3% (8/249)	70% (174/249)	88% (218/249)	12% (30/249)	30% (74/249)	NR	NR
	Only chemo			14% (36/250)	10% (23/238)	2% (5/250)	72% (181/250)	88% (221/250)	10% (24/250)	25% (63/250)	NR	NR
KEYNOTE-062 (17), global	Pembrolizumab (PD-1)	0.85; 95% CI, 0.70-1.03 (mOS: 12.5 m vs. 11.1 m)	0.84; 95% CI, 0.70-1.02 (mPFS: 6.9 m vs. 6.4 m)	49% (125/257); DOR: 6.8m	NR	7% (17/257)	66% (170/257)	95% (243/257)	NR	NR	NR	39% (99/257)
	Only chemo			37% (93/250); DOR: 6.8m	NR	8% (19/250)	72% (181/250)	94% (235/250)		NR	NR	36% (90/250)
CheckMate 649 (18, 23), global	Nivolumab (PD-1)	0.79; 95% CI, 0.71-0.88 (mOS: 13.7 m vs. 11.6 m)	0.79; 95% CI, 0.71-0.89 (mPFS: 7.8 m vs. 6.9 m)	58% (54%-62%); DOR: 8.5 m	17% (135/782)	3% (23/789)	70% (554/789)	96% (757/789)	16% (126/789)	81% (641 /789 )	60% (473/789 )	NR
	Only chemo			46% (13/249); DOR: 6.9 m	10% (77/767)	3% (21/792)	70% (556/792)	95% (756/792)	16% (127/792)	83% (655 /792 )	61% (482/792 )	NR
ORIENT-16 (19), China	Sintilimab (PD-1)	0.77; 95% CI, 0.63-0.94 (mOS: 15.2m vs. 12.3m)	0.64; 95% CI, 0.52-0.77 (mPFS: 7.1m vs. 5.7m)	58% (152/261); DOR: 8.4m	26%(86/328)	NR	81% (266/327)	91% (299/327)	NR	84% (275/ 327)	60% (197/327)	45% (146/ 327)
	Only chemo			48% (123/254); DOR: 5.5m	22% (70/320)	NR	81% (263/323)	93% (299/323)		84% (271/ 323)	62% (200/323)	44% (142/ 323)
ATTRACTION-4 (20), Asia	Nivolumab (PD-1)	0.90; 95% CI, 0.75-1.08 (mOS: 17.5m vs. 17.2m)	0.68; 95% CI, 0.51-0.90 (mPFS: 10.5m vs. 8.3m)	57% (208/362); DOR: 12.9m	19% (69/359)	NR	65% (237/362)	77% (280/362)	16% (58/362)	NR		
	Only chemo			48% (173/362); DOR: 8.7m	10% (35/358)	NR	66% (238/362)	77% (279/362)	15% (56/362)			
RATIONALE-305 (21), global	Tislelizumab (PD-1)	0.80; 95% CI, 0.70-0.92 (mOS: 15.0m vs. 12.9m)	0.78; 95% CI, 0.67-0.90 (mPFS: 6.9m vs. 6.2m)	47% (237/501); DOR: 8.7m	23% (113/498)	3%(16/501)	81% (405/501)	99% (494/501)	NR	55% (274/501)*		
	Only chemo			41% (201/496); DOR: 6.2m	15% (72/494)	5%(24/496)	80% (395/496)	99% (490/496)		55% (272/496)*		
KEYNOTE-859 (22), global	Pembrolizumab (PD-1)	0.78; 95% CI, 0.70-0.87 (mOS: 12.9m vs. 11.5m)	0.76; 95% CI, 0.67-0.85 (mPFS: 6.9m vs. 5.6m)	51% (405/790); DOR: 8.0m	23% (184/785)	5%(39/790)	81% (640/790)	96% (761/790)	NR	78% (618/ 790)	NR	35% (279/ 790)
	Only chemo			42% (331/789); DOR: 5.7m	19% (146/787)	4%(35/789)	76% (603/789)	96% (759/790)		78% (617/ 789)	NR	34% (272/ 789)

NR: Not reported; Chemo:Chemotherapy; HR: Hazard ratio; ITT: Intention-to-treat; sTRAE: Serious Treatment-related adverse event; CPS: Combined positive score; TAP: Tumor area positivity; MSI-H: Microsatellite instability-high; GC: Gastric cancer; ORR: Objective response rate; mOS: Median overall survival; OS: Overall survival; mPFS: Median progression-free survival; PFS: Progression-free survival; ICIs: Immune checkpoint inhibitors; m: Month; DOR: Duration of response.

*:PD-L1 expression TAP ≥5%.

#### 3.4.1 Overall survival

**Figure 4:**
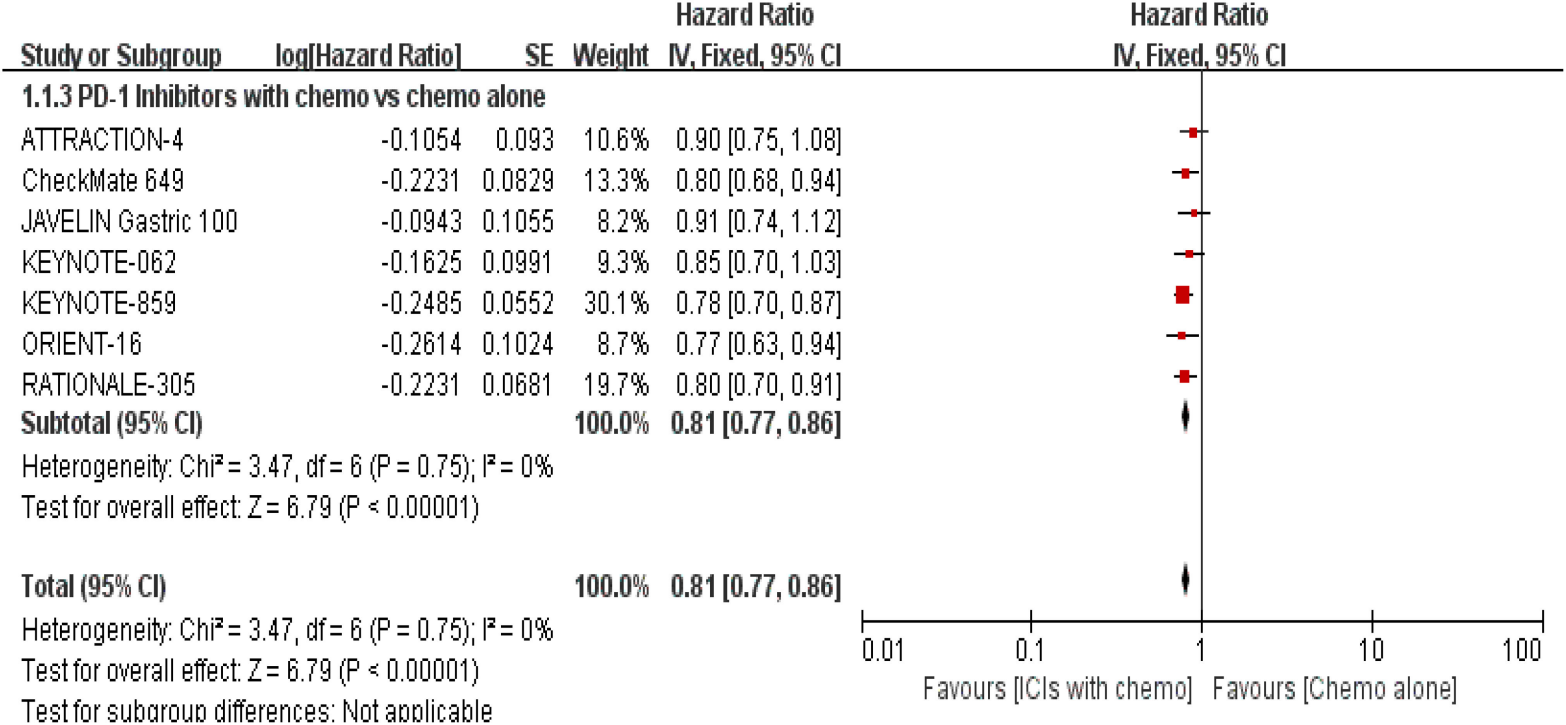
Forest plot for OS of all patients.

**Figure 5:**
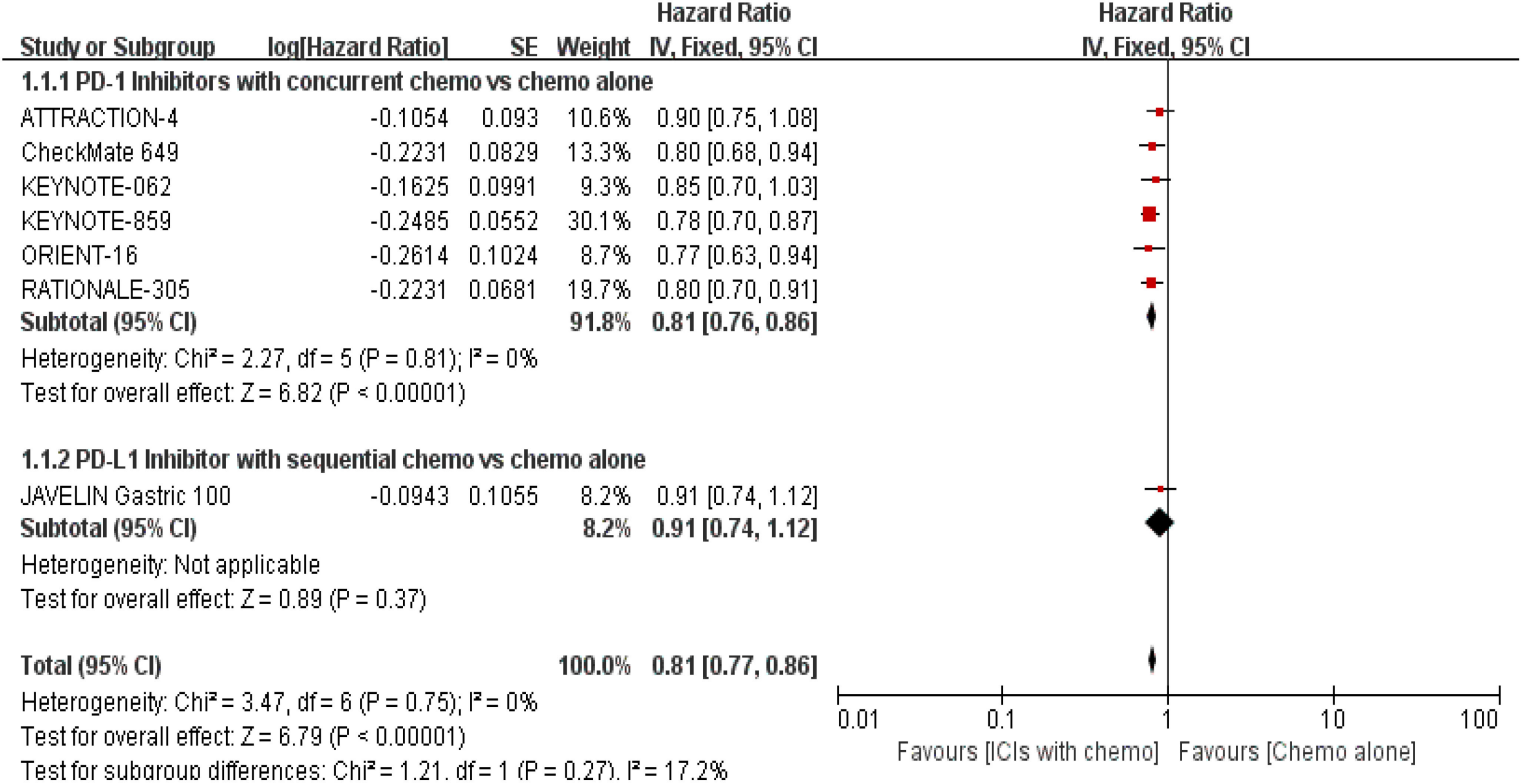
Forest plot for subgroup analysis of the OS (concurrent vs. sequential PD-1/PD-L1 inhibitors).

The 7 trials (16–22) that were included underwent OS comparisons. The inverse ANOVA with the Q-test for heterogeneity revealed no significant differences between the studies. According to the data, the experimental arm extended the OS in patients with advanced GC/GEJC when compared to the control arm [HR = 0.81, 95% CI (0.77, 0.86), P < 0.00001; I^2^ = 0%, thus a fixed-effect model was utilized]. The differences in immunotherapy regimens between the two arms in this investigation also prompted additional subgroup analysis, which showed an adjacent OS benefit in the sequential arm [HR = 0.91, 95% CI: (0.74–1.12), P = 0.37] in comparison with the concurrent arm. This could be largely attributed to the role of variations of treatment sequence, but not excluding the different mechanisms ICIs work. (**Figures 4**, **5**. illustrated the above-mentioned results).

#### 3.4.2 Progression-free survival

**Figure 6:**
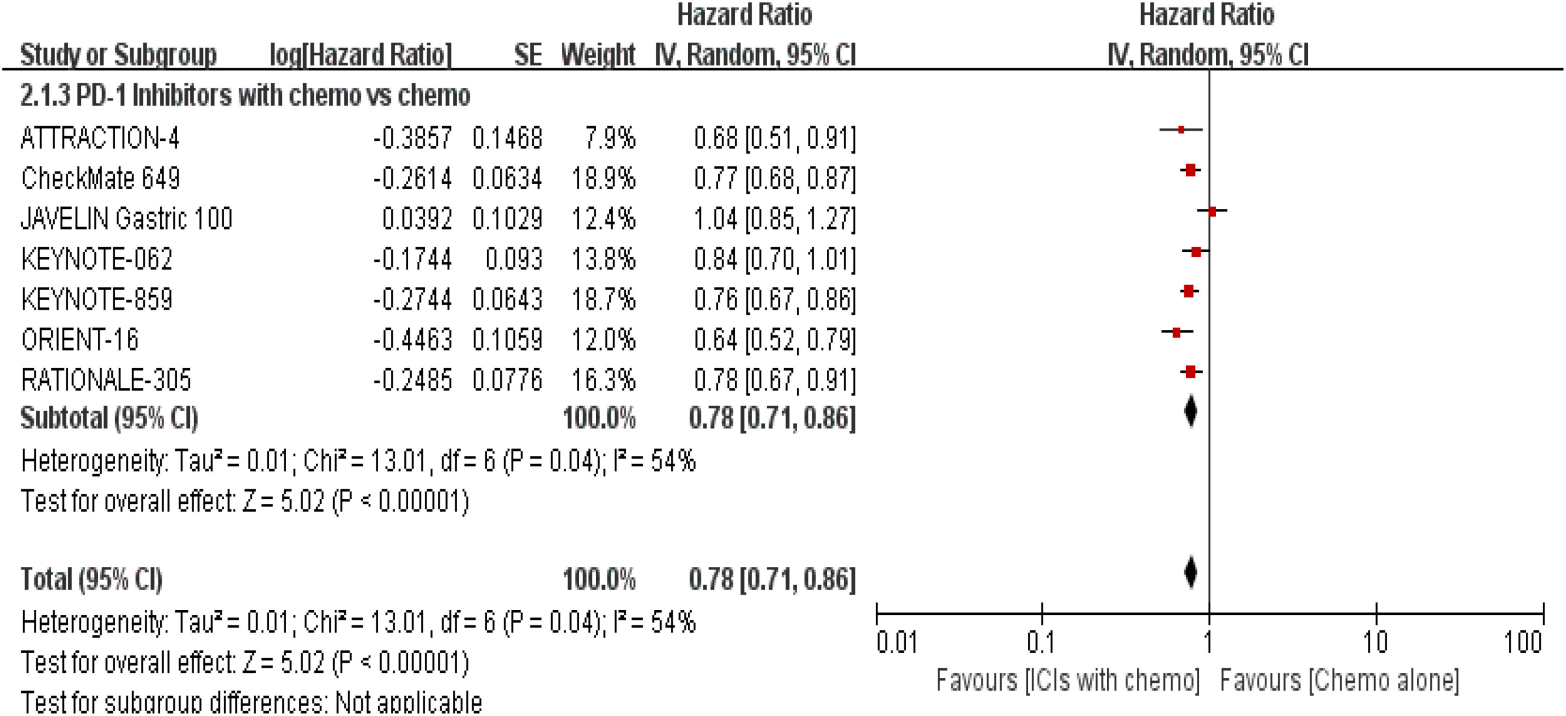
Forest plot for PFS of all patients.

**Figure 7:**
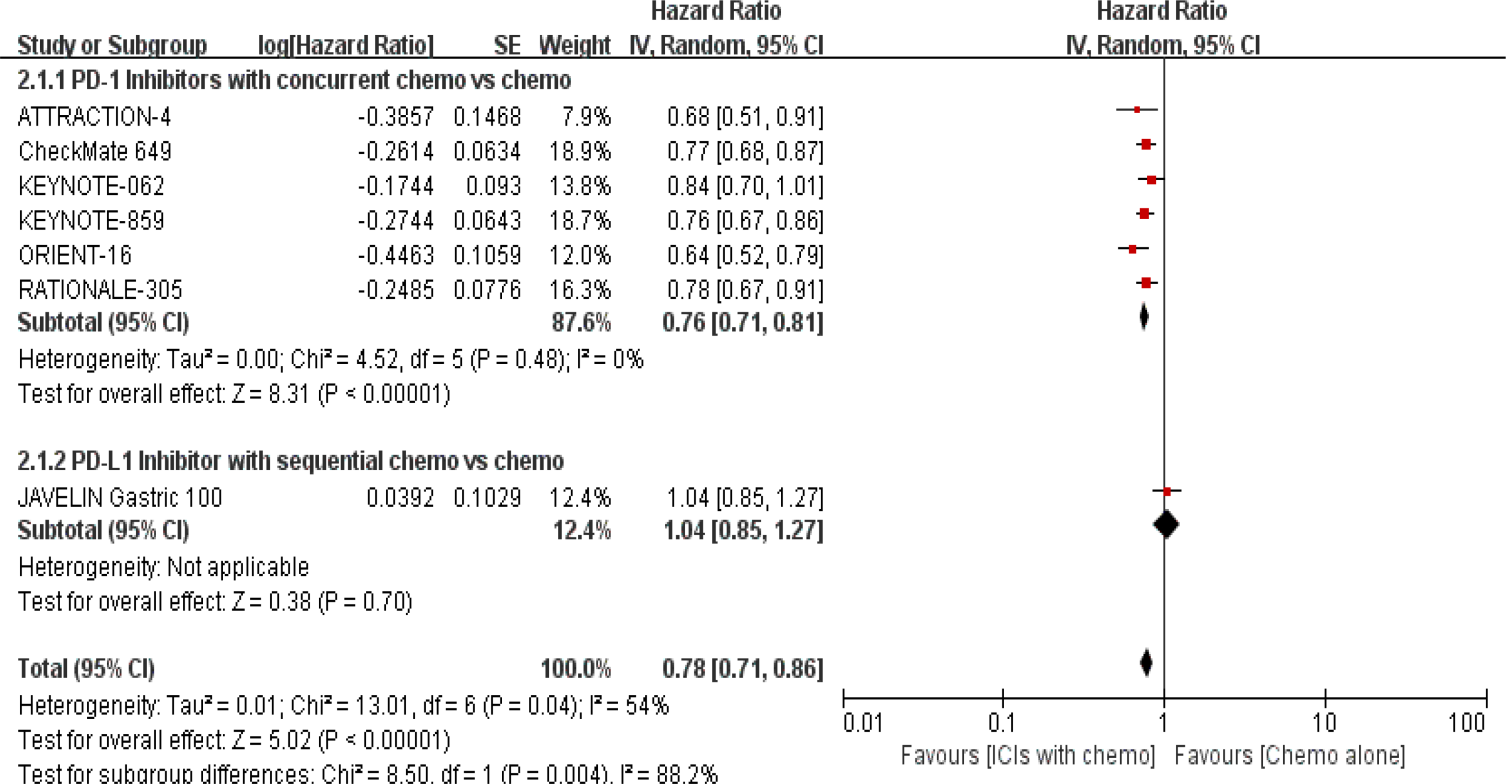
Forest plot for subgroup analysis of the PFS (concurrent vs. sequential PD-1/PD-L1 inhibitors).

PFS was documented in every experiment that was included (16–22). According to the previously indicated accessible RCTs, there was a statistically significant difference in PFS [RR = 0.78, 95% CI (0.71, 0.86), P < 0.00001; I^2^ = 54%; a random-effect model was used to minimize heterogeneity error, displayed in **Figures 6**, **7**]. Heterogeneity within each group has clearly decreased following a subgroup analysis and the removal of the sequential arm from the JAVELIN Gastric 100 trial (16). Additionally, the HR revealed a statistically significant effect [HR = 0.76, 95% CI: (0.71–0.81), P < 0.00001], indicating that immune-combination therapy is superior to chemo monotherapy in advanced gastric or gastroesophageal junction adenocarcinoma with HER-2 non-expression from the initial PFS results.

#### 3.4.3 Objective response rate

**Figure 8:**
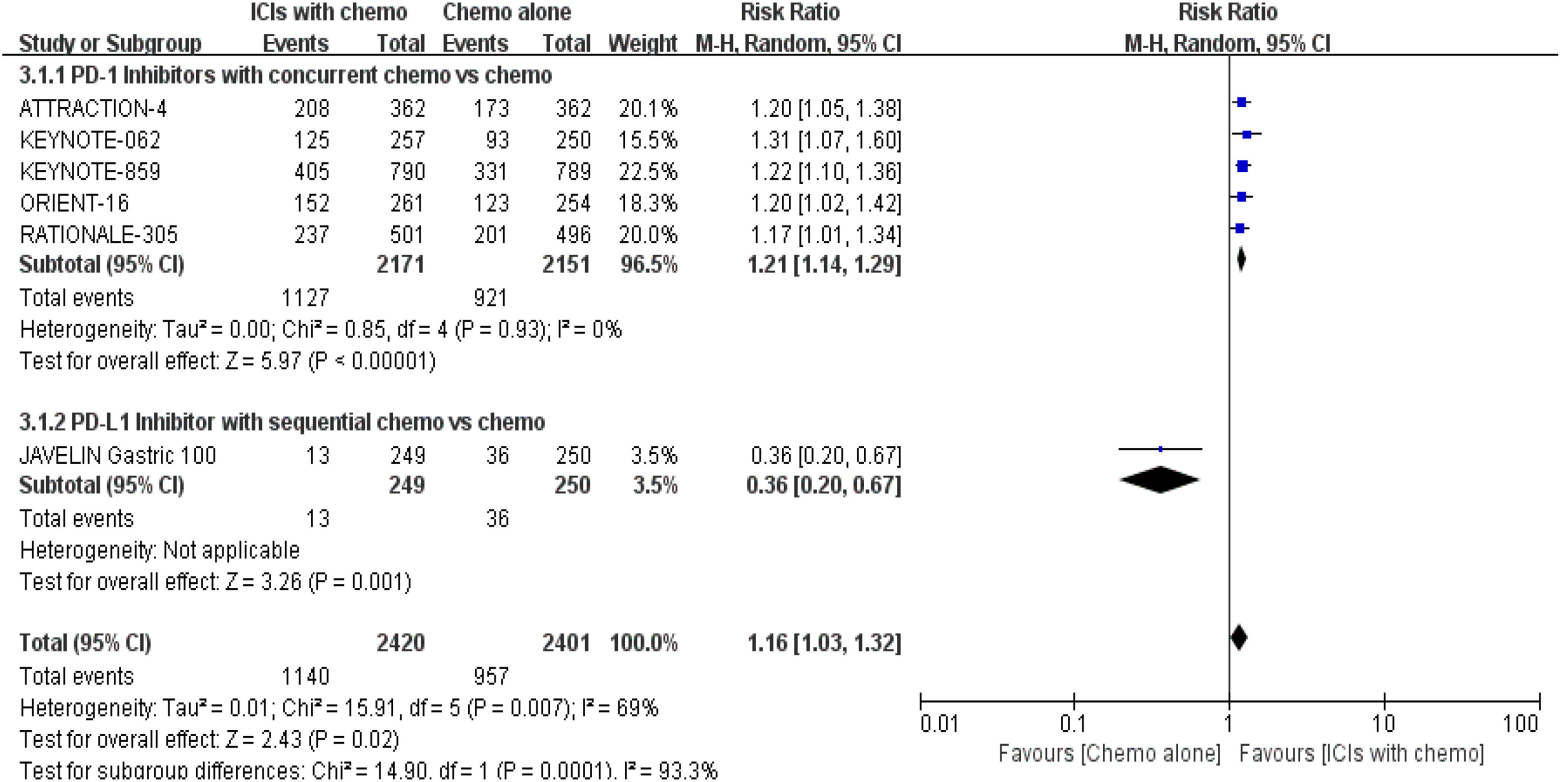
Forest plot for subgroup analysis of the ORR (concurrent vs. sequential PD-1/PD-L1 inhibitors).

PD-1/PD-L1 inhibitors plus chemo significantly increased ORR when compared to the control group, according to 6 RCTs (16, 17, 19–22) that reported ORR for evaluation [RR = 1.16, 95% CI (1.03, 1.32), P = 0.02]. Additionally, subgroup investigations were conducted based on variations in treatment regimens by the same methodology. The former (concurrent chemo) was preferred [RR = 1.21, 95% CI (1.14, 1.29), P < 0.00001; the prespecified analysis’s findings were shown in **Figure 8**].

### 3.5 Safety indicators: sTRAEs

**Figure 9:**
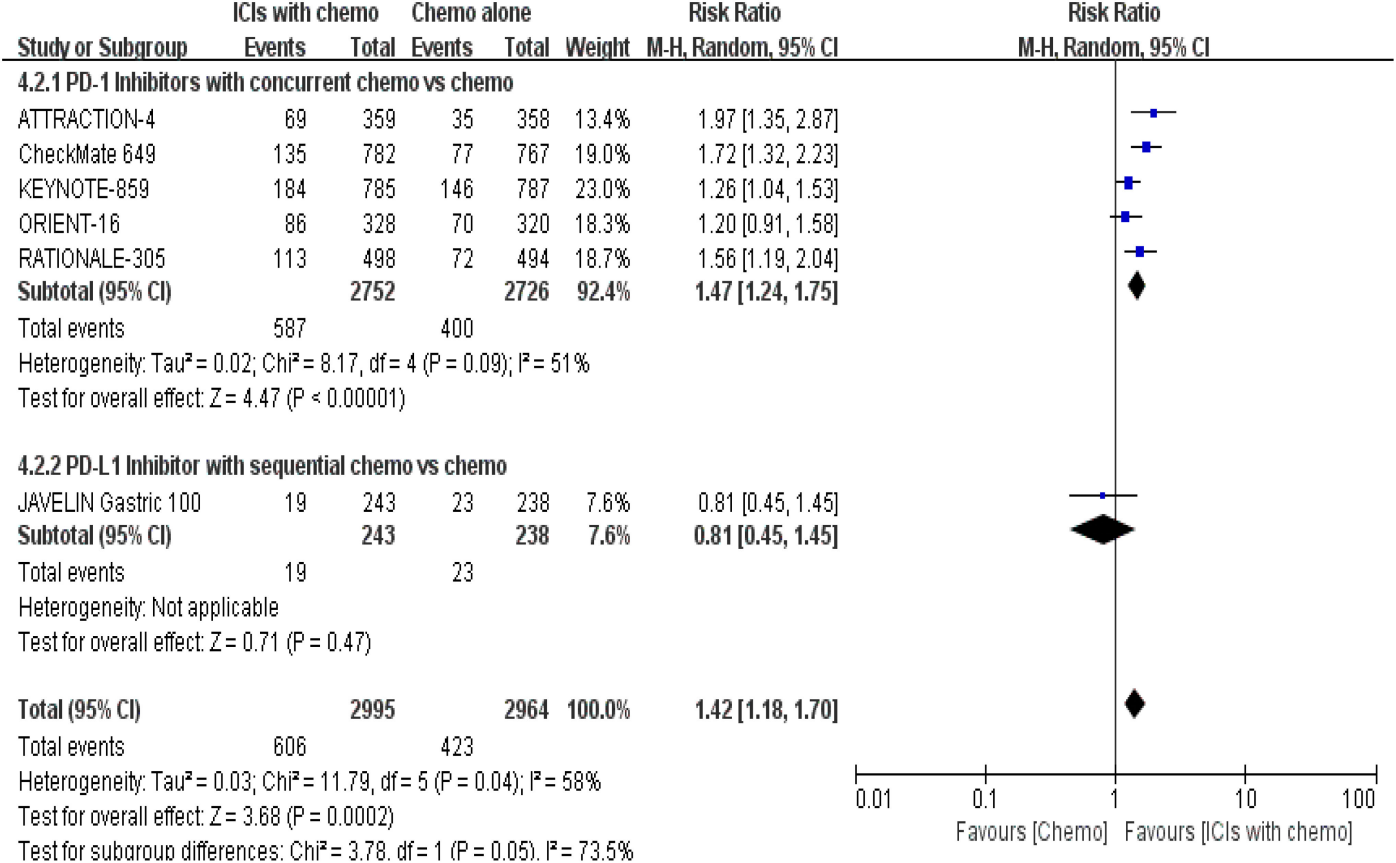
Forest plot for subgro**up analysis of the sTRAE rate (**concurrent vs. sequential PD-1/PD-L1 inhibitors).

5 RCTs (16, 18–22) reported sTRAEs rates for safety profile assessment; in the security analysis, statistically significant differences about sTRAEs were observed in the prespecified ICIs/chemo arm. Meanwhile, the synchronous therapy group was associated with higher incidences of sTRAEs [RR = 1.47, 95% CI (1.24, 1.75), P < 0.00001; a random-effect model was shown in **Figure 9**]; in the analysis of the subgroup, whereas the sequential treatment arm has not demonstrated that [RR = 0.81, 95% CI (0.45, 1.45), P = 0.47].

### 3.6 Subgroup analysis for the mOS

#### 3.6.1 GC or GEJC

**Figure 10:**
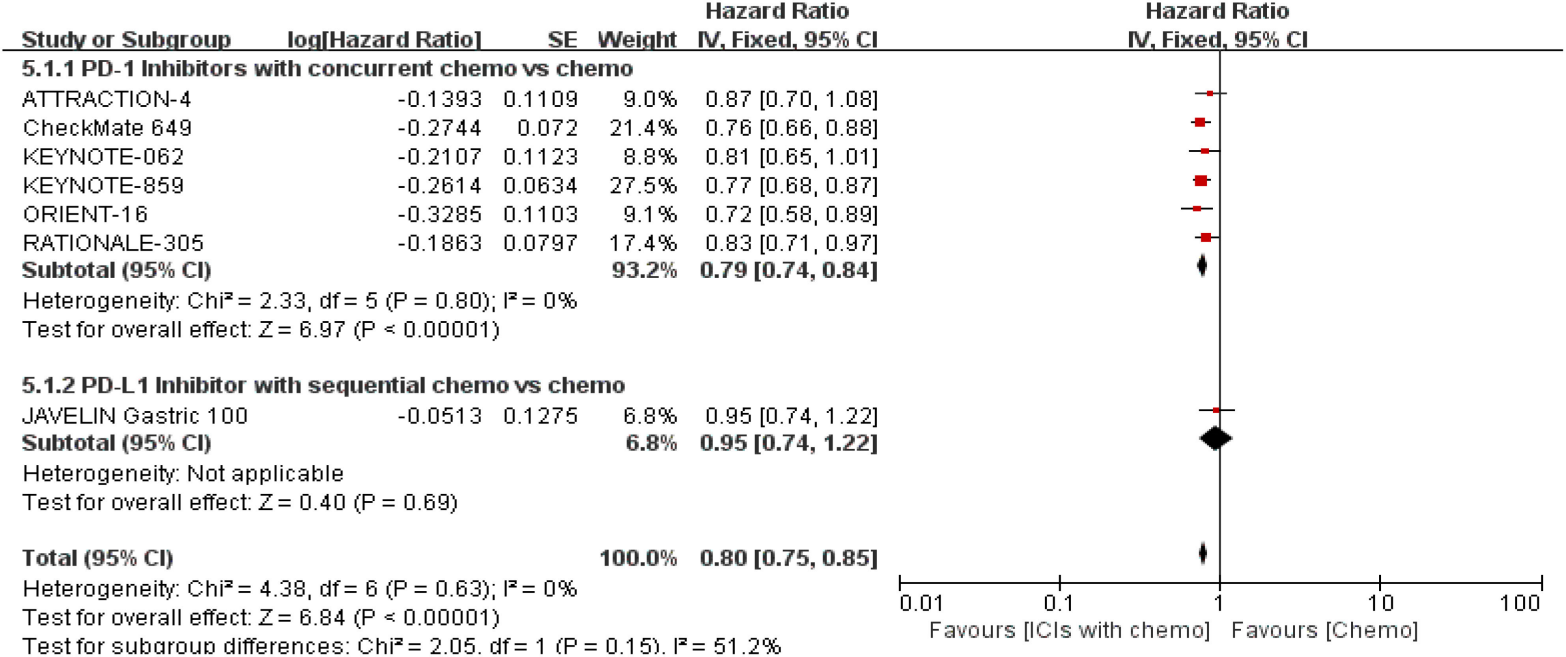
Forest plot for the median OS in the GC group.

**Figure 11:**
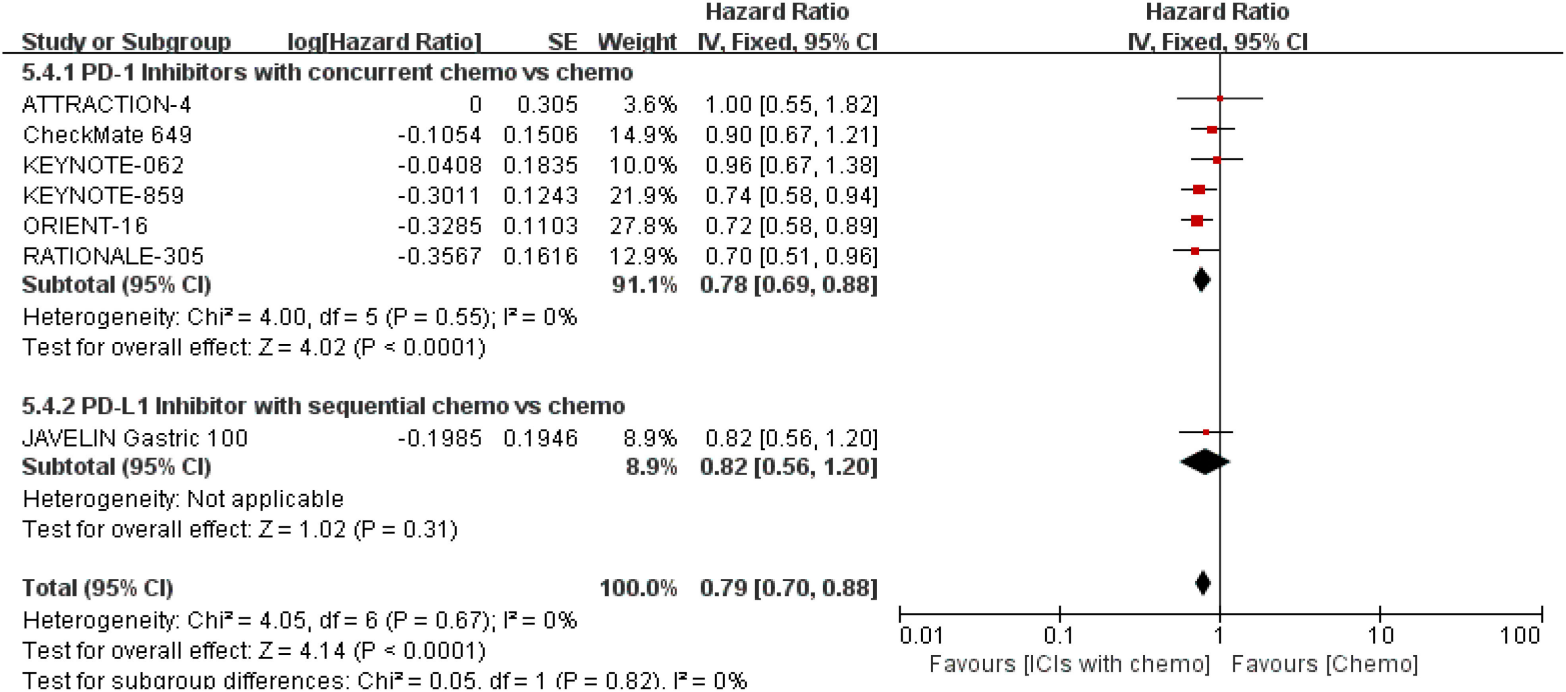
Forest plot for the median OS in the GEJC group.

7 trials (16–22) had HR values for GC or GEJC in the subgroup analysis of tumor site, which were displayed in **Figures 10**, **11**. The overall HRs were 0.80 (95% CI: 0.75-0.85; a p-value < 0.00001) for GC and 0.79 (95% CI: 0.70-0.88; a p-value < 0.0001) for GEJC individually. However, it seemed to be unclear why there was a junctional effect in the sequential study; likewise, the order of treatment is probably the main cause of these variations in results, and the role of ICIs cannot be completely ruled out. Indeed, our findings showed that PD-1/PD-L1 inhibitors plus chemo could decrease the HR values in both groups, but it’s just a different margin.

#### 3.6.2 Metastasis status

**Figure 12:**
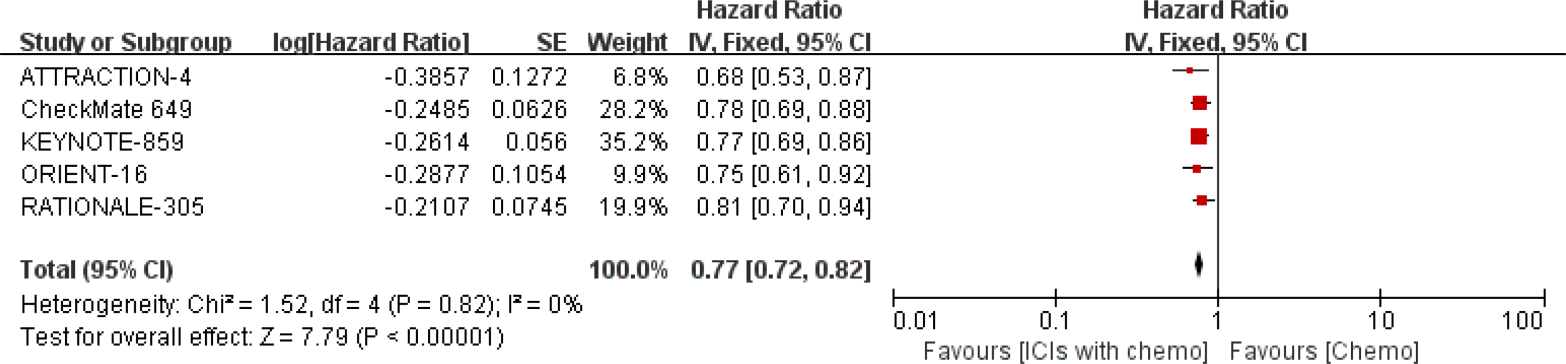
Forest plot for the median OS in the metastasis group.

We investigated the relationship between the presence of metastatic disease and mOS further with 5 RCTs (18–22). PD-1/PD-L1 inhibitors plus chemo demonstrated a significant decrease with the HR value in the metastasis status, being 0.77 (95% CI: 0.72-0.82; I^2^ = 0%, then a fixed effect model was used, shown in **Figure 12**).

#### 3.6.3 MSI-H group

**Figure 13:**
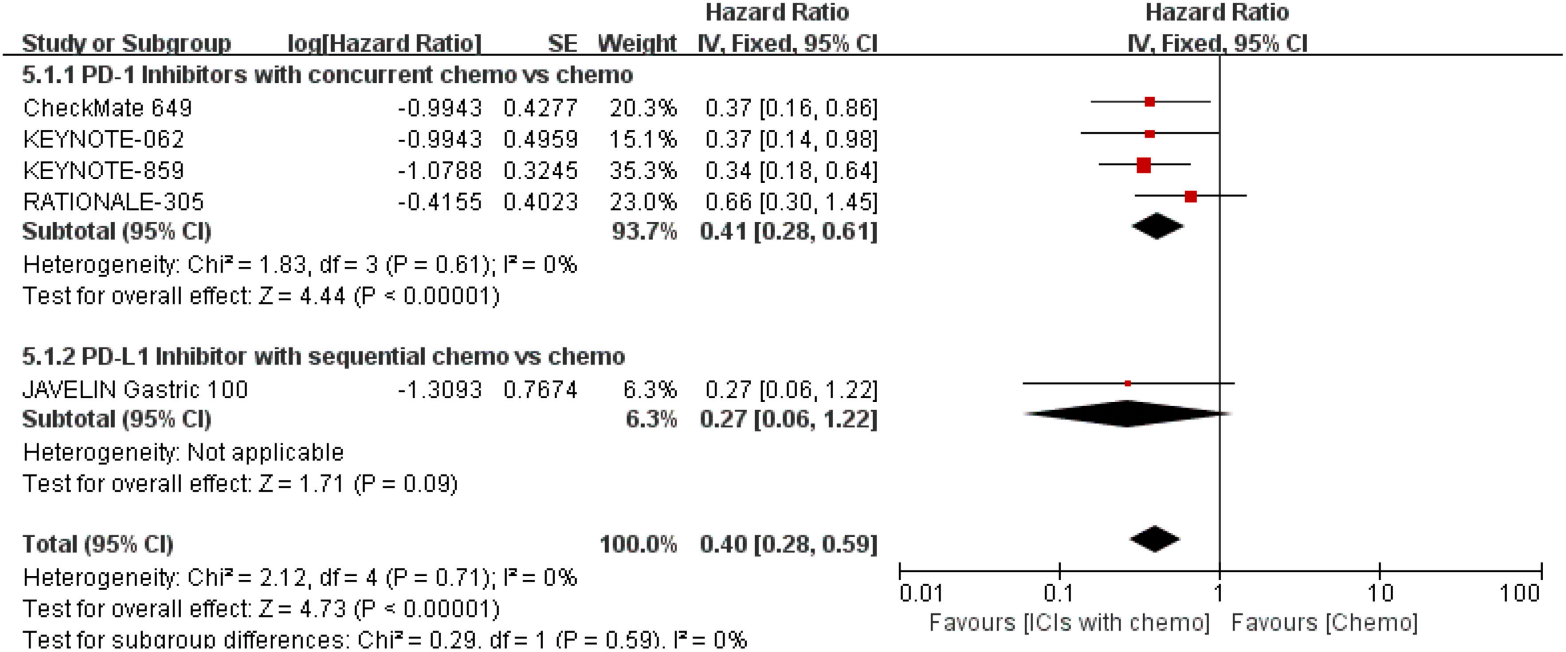
Forest plot for the median OS in the MSI-H group.

**Figure 14:**
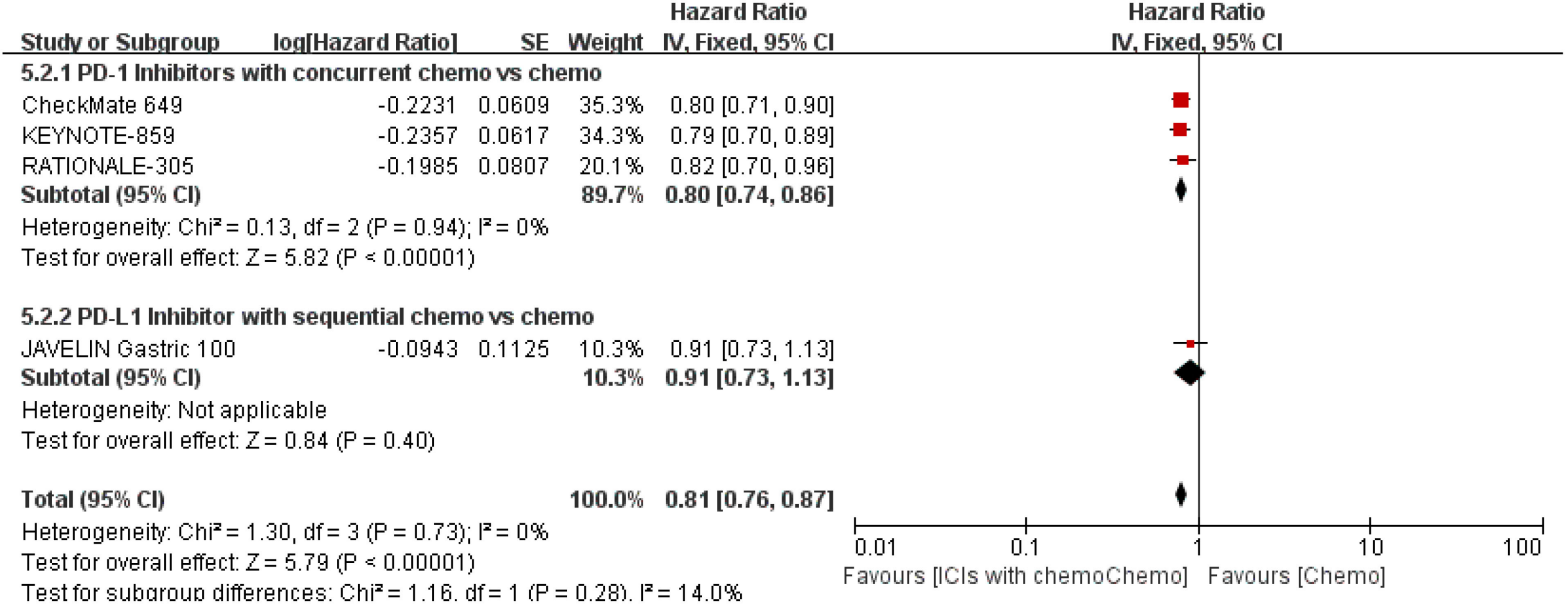
Forest plot for the median OS in the MSS group.

With p-values of 0.40 and 0.81, respectively, our subgroup analysis based on MSS status did discover a statistically significant effect in both the MSI-H and MSS arms (16–18, 21, 22). Compared with the MSS arm (HR: 0.80), ICIs with concurrent chemo regimens produce greater OS advantages in the MSI-H arm (HR: 0.41). Nevertheless, the mentioned OS variances between the MSI-H and MSS arms were barely affected by treatment sequence. Refer to **Figures 13**, **14** for specific details.

#### 3.6.4 Tumor cell PD-L1 expression

**Figure 15:**
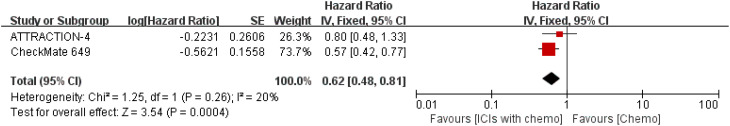
Forest plot for the median OS in the tumor cell PD-L1 expression ≥ 1% group.

The results of this investigation showed that higher PD-L1 expression was associated with a reduced HR of an event that occurred following treatment. The addition of PD-1/PD-L1 inhibitors based on the traditional chemo regimen decreased the risk of death in the subgroup by 38%, according to our meta-analysis founded on two trials (18, 20). For instance, we noticed that the PD-L1 expression of the tumors ≥1% group showed statistical significance with an HR of 0.62 (95% CI: 0.48-0.81, P = 0.0004; a fixed effect model was used, shown in **Figure 15**) between the two arms.

#### 3.6.5 PD-L1 CPS level

**Figure 16:**
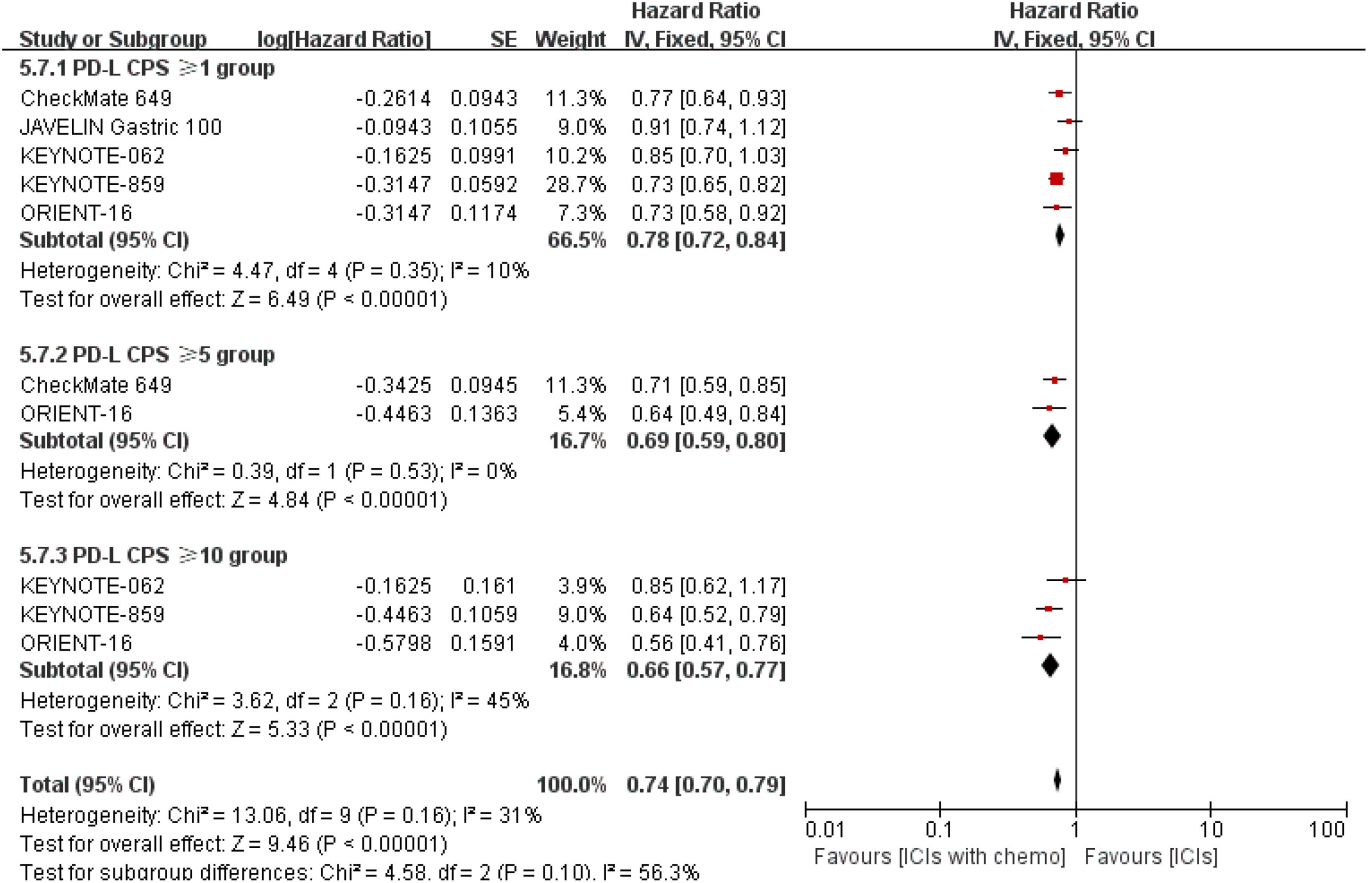
Forest plot for the median OS according to PD-L1 CPS level.

As for the subset investigation of PD-L1 CPS level, respectively reported in the included trials (16–19, 22), undoubtedly, there were statistically distinct discrepancies with all p-values of < 0.00001. The HR values were separately 0.78 and 0.69 for PD-L1 CPS of ≥ 1 and ≥ 5. Both groups favor PD-1/PD-L1 inhibitors with chemo, although there may be a higher probability of sTRAEs. Furthermore, the risk value was minimal in the PD-L1 CPS of ≥ 10 subgroup [HR = 0.66, 95% CI (0.57, 0.77); a p-value < 0.00001, shown in **Figure 16**].

## 4 Discussion

Advanced GC or GEJC patients have a dismal prognosis owing to the fact that standard chemo schemes show minor gains at treating these types of tumors, and no obvious development about treatment options has been made in the past decade. However, in recent years, numerous RCTs have demonstrated that adding ICIs to standard chemo regimens, a research hotspot and new treatment modality in the anti-tumor therapy of solid tumor fields, dramatically improves survival and alters the way the malignancies are treated without compromising the quality of life (QoL) (16–22). Therefore, immunotherapy has been considered a potential therapeutic approach that is frequently utilized as a supplement to conventional treatments (e.g., palliative surgery, radiation, and chemo) for those with advanced disease and negative HER-2 status. The immune checkpoint-mediated inhibitory signaling pathway allows tumor cells to evade the immunological response; hence, by blocking this immune escape pathway, ICIs can largely restore the immune response (24, 25). Researchers have placed a strong emphasis on the clinical use of immunotherapy in the treatment of advanced GC/GEJC patients, and plenty of major research has been carried out or is in progress; nevertheless, the effectiveness of PD-1/PD-L1 inhibitors in combination with chemo in advanced GC/GEJC is still controversial with respect to their magnitude of survival benefits according to populations with PD-L1 positivity cut-off value or degree of expression.

The JAVELIN Gastric 100 trial (16) showed that maintenance therapy with Avelumab did not show better OS and PFS benefits compared to chemo alone and was superior in terms of safety with a milder adverse event profile and prolonging duration of response (DOR) in the given subgroups. Due to the limitations of immunotherapy as a single agent, current clinical trials are shifting towards immunotherapy in combination with chemo strategies. When compared to standard regimens, this meta-analysis study revealed enhanced OS advantages in the combination group for patients with terminal GC/GEJC. According to the comparable results from the global KEYNOTE-859 study (22), the mOS (12.9 m vs. 11.5 m) and mPFS (6.9 m vs. 5.6 m) were longer in the pembrolizumab-treated plus chemo arm. In the same way, for the CheckMate-649 phase III study (18), all randomly assigned patients who received nivolumab in addition to chemo have benefited more than those who received chemo alone, with respective mOS of 13.7 m and 11.6 m (HR: 0.79). However, these trends were not found in the sequential treatment arm.

PFS is widely used as a surrogate endpoint for OS among patients with tumors. According to our study’s results, PFS was considerably gained in the synchronized arm as opposed to the chemo-only arm. The first PD-1 inhibitor to be utilized in Chinese patients with GC/GEJC is sintilimab, which was tested in the randomized, double-blind, phase III trial ORIENT-16 (19). Random assignment was executed to place 327 participants in the sintilimab-chemo group and 323 individuals in the placebo-chemo group. The two arms had mPFS of 7.1 m and 5.7 m, respectively (HR = 0.64), which resulted in a 36% reduction in the likelihood of disease progression, showing favorable short-term efficacy. Similarly, the above findings were not suitable for the sequential arm. Based on the version 1.1 content of the efficacy criteria for solid tumors, complete or partial remission was predefined as an effective treatment. According to the overall analysis and after excluding heterogeneous literature (17–22), the experimental group significantly outperformed the traditional chemo arm in terms of ORR for patients with advanced GC/GEJC when ICIs were adopted in conjunction with concurrent chemo (RR = 1.21, 95% CI: 1.14-1.29; P < 0.00001).

However, we couldn’t be entirely positive. In terms of treatment-related toxicity, the results showed that ICIs plus chemo may significantly contribute to more sTRAEs but have an acceptable safety and tolerability profile, with the most common comprising hematological and gastrointestinal toxicities and no unanticipated events (26). Hence, the beneficiary population should be accurately selected, and targeted interventions are required to prevent more serious complications from happening. Furthermore, cumulative data has shown that PD-L1 expression appears to be the most desirable predictive biomarker currently, and clinical trials have employed diverse approaches (including detection of antibodies to 73-10, 22C3, 28-8, and SP263 assays, among others) to assess PD-L1 status and different thresholds to define PD-L1 positivity, such as the tumor area positivity (TAP) score, tumor proportion score (TPS), and CPS (most commonly accepted clinically). In a *post hoc* analysis of the RATIONALE-305 trial, PD-L1 CPS and TAP scores were scored with high concordance among advanced GC/GEJC patients, especially when PD-L1 TAP scores were ≥ 5%. It is generally accepted that MSI-H, ≥ 1% PD-L1-positive tumor cells, and higher PD-L1 CPS groups are associated with a favorable prognosis. Both the ORIENT-16 trial (19) and the KEYNOTE-859 trial (22) obviously improved the mOS at a cutoff value of 10 or more with HRs of 0.64 and 0.56, respectively (both p-values < 0.00001). Similar survival data in the PD-L1 CPS of the ≥ 5 population was also noticed in the corresponding trials (CheckMate-649 (18, 23) and the recently published ORIENT-16 (19)). Additionally, the PD-L1 TAP score test was uniquely employed in the RATIONALE-305 trial (21). According to the final analysis, the tislelizumab combination chemo considerably increased the mOS in all randomized patients from 12.9 m to 15.0 m; the magnitude of improvement was considered statistically meaningful with risk reductions for 20% (HR = 0.80, 95% CI, 0.70-0.92; P = 0.001). With a mOS of 17.2 m in the pilot group, which was significantly superior to the 12.6 m in the placebo group (HR = 0.74, 95% CI, 0.59-0.94; P = 0.006), the survival outcomes in the prespecified PD-L1 TAP score of ≥ 5% subgroups were similarly stunning, accompanied by improvements in PFS and DOR.

PD-L1 CPS via assessment of both tumor and immune cells has shown better enrichment for efficacy evaluation, but under the implementation of this criteria, some subgroups of studies were still unable to benefit thoroughly from chemo-immunotherapy. The reason for that is the high degree of heterogeneity within GC/GEJC, such as different neoplasm sites and adjustments of intratumoral immune microenvironments. Besides, it’s unclear if PD-1/PD-L1 inhibitors could assist these patients who have a lower PD-L1 CPS (of < 5) to survive longer. Neither the ORIENT-16 nor CheckMate 649 trials published demonstrated statistically significant OS benefits in the subgroups with PD-L1 CPS of < 5. Therefore, a further division of the PD-L1 CPS of the < 5 population into the CPS of 1-4 or < 1 group may be necessary; the former are more likely to profit from dual therapy, while the latter are less likely to due to treatment-resistant “cold tumors” with immunity desert, as well as HER-2 positive patients with limited expression of HER-2 protein. Finally, due to the magnitude of OS benefits of anti-PD-1 therapy related to PD-L1 CPS status, we proposed an algorithm based on available evidence combining ICIs and chemo as a first-line treatment to reinforce advanced HER-2 negative patients with GC/GEJC who are inappropriate for surgery or who may benefit less from chemo alone, which was demonstrated in **Table 4**.

**Table 4:** Recommendations for first-line chemo combined with ICIs in HER-2 negative advanced GC/GEJC per the available evidence.

Study	CheckMate 649 (18, 23)	ORIENT-16 (19)	RATIONALE-305 (21)	KEYNOTE-859 (22)	GEMSTONE-303 (27, 28)	CPMPASSION-15 (29)*	Shen et al (30)**
Range	Global	China	Global	Global	China	China	China
ICIs	Nivolumab	Sintilimab	Tislelizumab	Pembrolizumab	Sugemalimab	Cadonilimab (AK104)	SHR-1701
CPS ≥ 10	+++ (mOS: 15.0 m vs. 10.9 m, HR: 0.66)	+++ (NR)	+++ (NR)	+++ (mOS: 15.7 m vs. 11.8 m, HR: 0.65)	+++ (mOS: 17.8 m vs. 12.5 m, HR: 0.64; mPFS: 7.8 m vs. 5.5 m, HR: 0.58)	+++ (NR)	+++ (NR)
CPS ≥ 5	+++ (mOS: 14.4 m vs. 11.1 m, HR: 0.71; mPFS: 8.3 m vs. 6.1 m, HR: 0.71; ORR: 60% vs. 45%; mDOR: 9.6 m vs. 7.0 m)	+++ (mOS: 18.4 m vs. 12.9 m, HR: 0.66; mPFS: 7.7 m vs. 5.8 m, HR: 0.63; ORR: 73% vs. 60%; DOR: 8.6 m vs. 5.5 m)	+++ (mOS: 16.4 m vs. 12.8 m, HR: 0.71)***	++ (NR)	+++ (mOS: 15.6 m vs. 12.6 m, HR: 0.75; mPFS: 7.6 m vs. 6.1 m, HR: 0.66; ORR: 69% vs. 53%; mDOR: 6.9 m vs. 4.6 m)	+++ (mOS: 15.3 m vs. 10.9 m, HR: 0.58)	+++ (mOS: 16.8 m vs. 10.4 m, HR: 0.53; mPFS: 7.6 vs. 5.5 m, HR: 0.52)
CPS 1-4	+ (NR)	+ (NR)	+ (NR)	++ (NR)	— (NR)	+++ (HR for mOS: 0.42-0.98)	++ (mOS: 15.8 m vs. 11.2 m, HR: 0.66; mPFS: 7.0 m vs. 5.5 m, HR: 0.57; both for the ITT population)
CPS < 1	— (NR)	— (NR)	— (NR)	— (NR)	— (NR)	+ (HR for mOS: 0.51-1.18)	— (NR)

ICIs, Immune checkpoint inhibitors; TAP, Tumor area positivity; mOS, Median overall survival; OS, Overall survival; mPFS, Median progression-free survival; PFS, Progression-free survival; m, Month; GC, Gastric cancer; GEJC, Gastroesophageal junction cancer; Chemo, Chemotherapy; m, month; NR, Not reported; HR, Hazard ratio; ORR, Objective response rate; ITT, Intention-to-treat; DOR, Duration of response.

+++: recommend highly ++: general recommendation +: discretionary recommendation -: not recommended.

CPMPASSION-15*: As the first phase III clinical trial of a PD-1/CTLA-4 bispecific antibody in combination with chemotherapy for the first-line treatment of HER-2 negative advanced GC/GEJC, the COMPASSION-15 study, including 610 patients from 75 centers, breaks the previous situation of limited efficacy of immunotherapy in patients with low or negative PD-L1 expression. With a median follow-up of 18.7 months, preliminary results show mOS and mPFS benefits (HRs were 0.66, 14.1 m vs. 11.1 m, and 0.53, 7.0 m vs. 5.3 m, respectively, both P < 0.001) across the entire population, even in PD-L1 low-expression subgroups with a CPS of < 5 (HRs were 0.70, P = 0.01, and 0.60, P < 0.001, respectively). The aforementioned milestone findings, however, have not yet been fully published in addition to the prespecified interim analysis.

Shen et al.**: This was a 2-part phase 3 study, and SHR-1701 was a bifunctional agent composed of an IgG4 mAb targeting PD-L1 fused with the extracellular domain of the TGF-β type II receptor (TGF-βIIR). The first line, SHR-1701 plus CAPOX, showed a statistically significant and clinically meaningful benefit in OS compared with placebo plus CAPOX in patients with HER2-negative GC or GEJC, both in the PD-L1 CPS ≥5 population and in the ITT population (15.8 m vs. 11.2 m, HR: 0.66, P < 0.0001) regardless of PD-L1 expression level, presenting as a new treatment option. Similarly, the above results were also not published in full but only presented as a mini oral session at ESMO Congress 2024.

***: PD-L1 expression TAP ≥5%.

## 5 Limitations

Unfortunately, there are several limitations to our meta-study worth being discussed. First, 7 RCTs (16–22) were included, indicating that there were a restricted number of advanced GC/GEJC sufferers involved; second, distinctions for age, ECOG performance, regions, PD-L1 expression cut-off values, and MSI detection standards among studies may have also affected the results; and third, even within RCTs, there were variations in the heterogeneity within gastric cancer populations, regimen completion, ICIs/chemo cycles, and duration of treatment time, which could have contributed to outcome errors. Lastly, given that separate PD-L1 expression might not be the best indicator, different biomarkers with predictive value for the efficiency of tumor immunotherapy are being investigated, and prediction models are being developed in the future (31–33). In addition, we did not conduct subgroup analysis further based on region, gender, and ECOG status, which may also be potentially favorable for screening the target population and achieving precision treatment. At the same time, we anticipate that further large-sample and multicenter trials worldwide will be available to address the previously mentioned confusing issues. Besides, given the beneficial short-term survival data from the CPMPASSION-15 trial (29) (employing PD-L/CTLA-4 antibody regardless of PD-L1 level) and Shen et al. (30) (adopting 1L SHR-1701 plus CAPOX) carried out in China, we also eagerly await the outcomes of the aforementioned study’s longer follow-up duration owing to the common delayed treatment effect with immunological drugs, particularly with low PD-L1 expression or even non-expression individuals treated by dual antibiotic drugs. As a simple example, the CheckMate 649 study (23) updated 5-year follow-up results, which continued to show mOS and mPFS benefits from nivolumab plus chemo in all randomly assigned patients (both HRs were 0.79), especially in patients with PD-L1 CPS ≥5 (HR values were better, both 0.71). However, regrettably, that study was left out of our meta-analysis given that we had submitted the manuscript to the Frontiers in Immunology editors about a month earlier prior to the GEMSTONE-303 (28) full-text investigation data being published in the journal *JAMA* (February 24, 2025).

## 6 Conclusions

Currently, ICIs drugs have been adopted extensively in Asia, Europe, and North America despite patients experiencing high levels of toxicity; in parallel, our study offers significant clinical evidence pertinent to the care of patients with advanced non-HER2-positive GC or GEJC worldwide, e.g., the level of evidence for treatment recommendations in **Table 4**. Generally speaking, participants respond better to concurrent leveraging immunochemotherapy when PD-L1 CPS cutoffs detected are higher (commonly PD-L1 CPS of ≥ 5) or microsatellite instability-high tumors are recruited since consistent OS benefits among these subsets were also observed in the RATIONALE-305 (21), CheckMate 649 (18, 23), and ORIENT-16 (19) studies, with observed declining HRs indicating enrichment of superior OS. In addition, since PD-L1 expression and PD-L1 CPS (or TAP score) vary widely from pre- to post-treatment due to the spatiotemporal heterogeneity of GC/GEJC and PD-L1 detection results being limited by the incoherence of specimen collection handling and analytical concordance of test assays and laboratory platforms, the prognostic value for PD-L1 assays is questioned by certain scholars and may be evaluated in harmonization standards or conjunction with other biomarker assessments, e.g., TMB-H, CLDN18.2, NTRK, and FGFR2 targets, or clinical features (34). Numerous preclinical and clinical research studies (35–37) have provided evidence of the remote effects of radiotherapy. By encouraging the generation of tumor antigens and the cross-presentation of tumor-derived antigens to T cells, in addition to killing tumor cells with irradiation, it can boost anti-tumor adaptive immunity. For instance, Wei et al. (38) reported the findings of a prospective, phase II multicenter trial that demonstrated the effectiveness of sintilimab, a PD-1 inhibitor, as a neoadjuvant treatment option for locally advanced GC/GEJC when combined with concurrent chemoradiotherapy. This “triple-strike” treatment produced a 38.2% pCR rate, which opens up new avenues for advanced cancer research.

In conclusion, although immunotherapy has changed the treatment paradigm for GC/GEJC, some patients may still have limited benefits due to immunoresistance. The uncovering of novel targets (e.g., VEGFR inhibitors, etc.) may further support individual stratified precision therapy due to the identification of the primary or secondary immunoresistant subgroups enriching the immunotherapeutically beneficial populations at the initial lines (39, 40).

## Data Availability

The original contributions presented in the study are included in the article/supplementary material. Further inquiries can be directed to the corresponding authors.

## Glossary

ICIs: Immune checkpoint inhibitors
Chemo: Chemotherapy
GC: Gastric cancer
GEJC: Gastroesophageal junction cancer
RCTs: Randomized controlled trials
m: Months
RR: Risk ratio
CI: Confidence intervals
PRISMA: Preferred Reporting Items for Systematic Reviews and Meta-Analyses
PICOS: Population, intervention, comparison, outcomes, and study
MeSH: Medical subject heading
mOS: Median overall survival
OS: Overall survival
PFS: Progression-free survival
mPFS: Median progression-free survival
ORR: Objective response rate
CTCAE: National Cancer Institute Common Terminology Criteria for Adverse Events
MSI: Microsatellite instability status
CPS: Combined positive scores
PD-1: Programmed cell death protein-1
PD-L1: Programmed cell death protein ligand 1
HER2: Human epidermal growth factor receptor 2
FDA: Food and Drug Administration
NMPA: National Medical Products Administration
TAP: Tumor area positivity
TPS: Tumor proportion score
QoL: Quality of life
ICIs: Immune checkpoint inhibitors
HR: Hazard ratio
ECOG: Eastern Cooperative Oncology Group
MSI-H/dMMR: Microsatellite instability/mismatch repair deficiency
TMB-H: High tumor mutation burden
DOR: Duration of response
sTRAEs: Serious treatment-related adverse events
95% CI: 95% confidence interval.

## References

[1] Bray F Laversanne M Sung H Ferlay J Siegel RL Soerjomataram I . Global cancer statistics 2022: GLOBOCAN estimates of incidence and mortality worldwide for 36 cancers in 185 countries. CA Cancer J Clin. (2024) 74:229–63. doi: 10.3322/caac.21834 38572751

[2] Morgan E Arnold M Camargo MC Gini A Kunzmann AT Matsuda T . The current and future incidence and mortality of gastric cancer in 185 countries, 2020-40: A population-based modelling study. EClinicalMedicine. (2022) 47:101404. doi: 10.1016/j.eclinm.2022.101404 35497064 PMC9046108

[3] Cunningham D Allum WH Stenning SP Thompson JN Van de Velde CJ Nicolson M . Perioperative chemotherapy versus surgery alone for resectable gastroesophageal cancer. N Engl J Med. (2006) 355:11–20. doi: 10.1056/NEJMoa055531 16822992

[4] Chen ZD Zhang PF Xi HQ Wei B Chen L Tang Y . Recent advances in the diagnosis, staging, treatment, and prognosis of advanced gastric cancer: A literature review. Front Med (Lausanne). (2021) 8:744839. doi: 10.3389/fmed.2021.744839 34765619 PMC8575714

[5] Ychou M Boige V Pignon JP Conroy T Bouché O Lebreton G . Perioperative chemotherapy compared with surgery alone for resectable gastroesophageal adenocarcinoma: an FNCLCC and FFCD multicenter phase III trial. J Clin Oncol. (2011) 29:1715–21. doi: 10.1200/JCO.2010.33.0597 21444866

[6] Cao W Chen HD Yu YW Li N Chen WQ . Changing profiles of cancer burden worldwide and in China: a secondary analysis of the global cancer statistics 2020. Chin Med J (Engl). (2021) 134:783–91. doi: 10.1097/CM9.0000000000001474 PMC8104205 33734139

[7] Wei SC Duffy CR Allison JP . Fundamental mechanisms of immune checkpoint blockade therapy. Cancer Discovery. (2018) 8:1069–86. doi: 10.1158/2159-8290.CD-18-0367 30115704

[8] Shitara K Bang YJ Iwasa S Sugimoto N Ryu MH Sakai D . Trastuzumab deruxtecan in previously treated HER2-positive gastric cancer. N Engl J Med. (2020) 382:2419–30. doi: 10.1056/NEJMoa2004413 32469182

[9] Van Cutsem E di Bartolomeo M Smyth E Chau I Park H Siena S . Trastuzumab deruxtecan in patients in the USA and Europe with HER2-positive advanced gastric or gastroesophageal junction cancer with disease progression on or after a trastuzumab-containing regimen (DESTINY-Gastric02): primary and updated analyses from a single-arm, phase 2 study. Lancet Oncol. (2023) 24:744–56. doi: 10.1016/S1470-2045(23)00215-2 PMC11298287 37329891

[10] Bang YJ Van Cutsem E Feyereislova A Chung HC Shen L Sawaki A . Trastuzumab in combination with chemotherapy versus chemotherapy alone for treatment of HER2-positive advanced gastric or gastro-oesophageal junction cancer (ToGA): a phase 3, open-label, randomised controlled trial. Lancet. (2010) 376:687–97. doi: 10.1016/S0140-6736(10)61121-X 20728210

[11] Gandhi L Rodríguez-Abreu D Gadgeel S Esteban E Felip E De Angelis F . Pembrolizumab plus chemotherapy in metastatic non-small-cell lung cancer. N Engl J Med. (2018) 378:2078–92. doi: 10.1056/NEJMoa1801005 29658856

[12] Tallón de Lara P Cecconi V Hiltbrunner S Yagita H Friess M Bode B . Gemcitabine synergizes with immune checkpoint inhibitors and overcomes resistance in a preclinical model and mesothelioma patients. Clin Cancer Res. (2018) 24:6345–54. doi: 10.1158/1078-0432.CCR-18-1231 30154226

[13] Choueiri TK Motzer RJ Rini BI Haanen J Campbell MT Venugopal B . Updated efficacy results from the JAVELIN Renal 101 trial: first-line avelumab plus axitinib versus sunitinib in patients with advanced renal cell carcinoma. Ann Oncol. (2020) 31:1030–9. doi: 10.1016/j.annonc.2020.04.010 PMC8436592 32339648

[14] Jiang X Wang J Deng X Xiong F Ge J Xiang B . Role of the tumor microenvironment in PD-L1/PD-1-mediated tumor immune escape. Mol Cancer. (2019) 18:10. doi: 10.1186/s12943-018-0928-4 30646912 PMC6332843

[15] Huang L Yin Y Yang L Wang C Li Y Zhou Z . Comparison of antibiotic therapy and appendectomy for acute uncomplicated appendicitis in children: A meta-analysis. JAMA Pediatr. (2017) 171:426–34. doi: 10.1001/jamapediatrics.2017.0057 PMC5470362 28346589

[16] Moehler M Dvorkin M Boku N Özgüroğlu M Ryu MH Muntean AS . Phase III trial of avelumab maintenance after first-line induction chemotherapy versus continuation of chemotherapy in patients with gastric cancers: results from JAVELIN gastric 100. J Clin Oncol. (2021) 39:966–77. doi: 10.1200/JCO.20.00892 PMC8078426 33197226

[17] Shitara K Van Cutsem E Bang YJ Fuchs C Wyrwicz L Lee KW . Efficacy and safety of pembrolizumab or pembrolizumab plus chemotherapy vs chemotherapy alone for patients with first-line, advanced gastric cancer: the KEYNOTE-062 phase 3 randomized clinical trial. JAMA Oncol. (2020) 6:1571–80. doi: 10.1001/jamaoncol.2020.3370 PMC7489405 32880601

[18] Janjigian YY Shitara K Moehler M Garrido M Salman P Shen L . First-line nivolumab plus chemotherapy versus chemotherapy alone for advanced gastric, gastro-oesophageal junction, and oesophageal adenocarcinoma (CheckMate 649): a randomised, open-label, phase 3 trial. Lancet. (2021) 398:27–40. doi: 10.1016/S0140-6736(21)00797-2 34102137 PMC8436782

[19] Xu J Jiang H Pan Y Gu K Cang S Han L . Sintilimab plus chemotherapy for unresectable gastric or gastroesophageal junction cancer: the ORIENT-16 randomized clinical trial. JAMA. (2023) 330:2064–74. doi: 10.1001/jama.2023.19918 PMC10698618 38051328

[20] Kang YK Chen LT Ryu MH Oh DY Oh SC Chung HC . Nivolumab plus chemotherapy versus placebo plus chemotherapy in patients with HER2-negative, untreated, unresectable advanced or recurrent gastric or gastro-oesophageal junction cancer (ATTRACTION-4): a randomised, multicentre, double-blind, placebo-controlled, phase 3 trial. Lancet Oncol. (2022) 23:234–47. doi: 10.1016/S1470-2045(21)00692-6 35030335

[21] Qiu MZ Oh DY Kato K Arkenau T Tabernero J Correa MC . Tislelizumab plus chemotherapy versus placebo plus chemotherapy as first line treatment for advanced gastric or gastro-oesophageal junction adenocarcinoma: RATIONALE-305 randomised, double blind, phase 3 trial. BMJ. (2024) 385:e078876. doi: 10.1136/bmj-2023-078876 38806195

[22] Rha SY Oh DY Yañez P Bai Y Ryu MH Lee J . Pembrolizumab plus chemotherapy versus placebo plus chemotherapy for HER2-negative advanced gastric cancer (KEYNOTE-859): a multicentre, randomised, double-blind, phase 3 trial. Lancet Oncol. (2023) 24:1181–95. doi: 10.1016/S1470-2045(23)00515-6 37875143

[23] Yelena J Markus M Jaffer A Lin S Marcelo G Carlos G . Nivolumab (NIVO) + chemotherapy (chemo) vs chemo as first-line (1L) treatment for advanced gastric cancer/gastroesophageal junction cancer/esophageal adenocarcinoma (GC/GEJC/EAC): 5-year (y) follow-up results from CheckMate 649. J Clin Oncol. (2025) 43. doi: 10.1200/JCO.2025.43.4_suppl.398

[24] Marin-Acevedo JA Kimbrough EO Lou Y . Next generation of immune checkpoint inhibitors and beyond. J Hematol Oncol. (2021) 14:45. doi: 10.1186/s13045-021-01056-8 33741032 PMC7977302

[25] Sun Q Hong Z Zhang C Wang L Han Z Ma D . Immune checkpoint therapy for solid tumours: clinical dilemmas and future trends. Signal Transduct Target Ther. (2023) 8:320. doi: 10.1038/s41392-023-01522-4 37635168 PMC10460796

[26] Wang BC Zhang ZJ Fu C Wang C . Efficacy and safety of anti-PD-1/PD-L1 agents vs chemotherapy in patients with gastric or gastroesophageal junction cancer: a systematic review and meta-analysis. Med (Baltimore). (2019) 98:e18054. doi: 10.1097/MD.0000000000018054 PMC6882659 31764833

[27] Zhang X Wang J Wang G Zhang Y Fan Q Chuangxin L . GEMSTONE-303: Prespecified progression-free survival (PFS) and overall survival (OS) final analyses of a phase III study of sugemalimab plus chemotherapy vs placebo plus chemotherapy in treatment-naïve advanced gastric or gastroesophageal junction (G/GEJ) adenocarcinoma. Ann Oncol. (2023) 34:S1319. doi: 10.1016/j.annonc.2023.10.080

[28] Zhang X Wang J Wang G Zhang Y Fan Q Lu C . First-line sugemalimab plus chemotherapy for advanced gastric cancer: the GEMSTONE-303 randomized clinical trial. JAMA. (2025):e2428463. doi: 10.1001/jama.2024.28463 39992668 PMC11851304

[29] Shen L Zhang Y Li Z Zhang X Gao X Liu B . First-line cadonilimab plus chemotherapy in HER2-negative advanced gastric or gastroesophageal junction adenocarcinoma: a randomized, double-blind, phase 3 trial. Nat Med. (2025). doi: 10.1038/s41591-024-03450-4 39843940

[30] Peng Z Wang J Zhang Y Li H Zhao Q Zhu X . Phase III study of SHR-1701 versus placebo in combination with chemo as first-line (1L) therapy for HER2-negative gastric/gastroesophageal junction adenocarcinoma (G/GEJA). Ann Oncol. (2024) 35:1–72. doi: 10.1016/annonc/annonc1623

[31] Kim ST Cristescu R Bass AJ Kim KM Odegaard JI Kim K . Comprehensive molecular characterization of clinical responses to PD-1 inhibition in metastatic gastric cancer. Nat Med. (2018) 24:1449–58. doi: 10.1038/s41591-018-0101-z 30013197

[32] Llosa NJ Cruise M Tam A Wicks EC Hechenbleikner EM Taube JM . The vigorous immune microenvironment of microsatellite instable colon cancer is balanced by multiple counter-inhibitory checkpoints. Cancer Discovery. (2015) 5:43–51. doi: 10.1158/2159-8290.CD-14-0863 25358689 PMC4293246

[33] Yap DWT Leone AG Wong NZH Zhao JJ Tey JCS Sundar R . Effectiveness of immune checkpoint inhibitors in patients with advanced esophageal squamous cell carcinoma: A meta-analysis including low PD-L1 subgroups. JAMA Oncol. (2023) 9:215–24. doi: 10.1001/jamaoncol.2022.5816 PMC9857522 36480211

[34] Song J Zhu J Jiang Y Guo Y Liu S Qiao Y . Advancements in immunotherapy for gastric cancer: Unveiling the potential of immune checkpoint inhibitors and emerging strategies. Biochim Biophys Acta Rev Cancer. (2025) 1880:189277. doi: 10.1016/j.bbcan.2025.189277 39938663

[35] Galluzzi L Aryankalayil MJ Coleman CN Formenti SC . Emerging evidence for adapting radiotherapy to immunotherapy. Nat Rev Clin Oncol. (2023) 20:543–57. doi: 10.1038/s41571-023-00782-x 37280366

[36] Marciscano AE Walker JM McGee HM Kim MM Kunos CA Monjazeb AM . Incorporating Radiation Oncology into Immunotherapy: proceedings from the ASTRO-SITC-NCI immunotherapy workshop. J Immunother Cancer. (2018) 6:6. doi: 10.1186/s40425-018-0317-y 29375032 PMC5787916

[37] Colton M Cheadle EJ Honeychurch J Illidge TM . Reprogramming the tumour microenvironment by radiotherapy: implications for radiotherapy and immunotherapy combinations. Radiat Oncol. (2020) 15:254. doi: 10.1186/s13014-020-01678-1 33148287 PMC7640712

[38] Wei J Lu X Liu Q Fu Y Liu S Zhao Y . Neoadjuvant sintilimab in combination with concurrent chemoradiotherapy for locally advanced gastric or gastroesophageal junction adenocarcinoma: a single-arm phase 2 trial. Nat Commun. (2023) 14:4904. doi: 10.1038/s41467-023-40480-x 37580320 PMC10425436

[39] Rizvi N Ademuyiwa FO Cao ZA Chen HX Ferris RL Goldberg SB . Society for Immunotherapy of Cancer (SITC) consensus definitions for resistance to combinations of immune checkpoint inhibitors with chemotherapy. J Immunother Cancer. (2023) 11:e005920. doi: 10.1136/jitc-2022-005920 36918220 PMC10016262

[40] Routy B Le Chatelier E Derosa L Duong CPM Alou MT Daillère R . Gut microbiome influences efficacy of PD-1-based immunotherapy against epithelial tumors. Science. (2018) 359:91–7. doi: 10.1126/science.aan3706 29097494

